# Genome sequence of a European *Diplocarpon coronariae* strain and *in silico* structure of the mating-type locus

**DOI:** 10.3389/fpls.2024.1437132

**Published:** 2024-10-18

**Authors:** Sophie Richter, Sabine Kind, Thomas Oberhänsli, Michael Schneider, Natalia Nenasheva, Katharina Hoff, Jens Keilwagen, Il-Kweon Yeon, Vincent Philion, Shigeki Moriya, Henryk Flachowsky, Andrea Patocchi, Thomas Wolfgang Wöhner

**Affiliations:** ^1^ Institute for Breeding Research on Fruit Crops, Julius Kühn-Institute (JKI) – Federal Research Centre for Cultivated Plants, Dresden, Germany; ^2^ Institute for Plant Genetics, Leibniz University Hannover, Hanover, Germany; ^3^ Institute for Plant Protection in Fruit Crops and Viticulture, Julius Kühn Institute (JKI) Federal Research Centre for Cultivated Plants, Dossenheim, Germany; ^4^ Department of Crop Sciences, Research Institute of Organic Agriculture (FiBL), Frick, Switzerland; ^5^ Institute of Mathematics and Computer Science and Center for Functional Genomics of Microbes, University of Greifswald, Greifswald, Germany; ^6^ Institute for Biosafety in Plant Biotechnology, Julius Kühn-Institute (JKI) – Federal Research Centre for Cultivated Plants, Quedlinburg, Germany; ^7^ Gyeongsangbuk-do Agricultural Research and Extension Services (GBARES), Daegu, Republic of Korea; ^8^ Research and Development Institute for the Agri-Environment (IRDA), Québec, QC, Canada; ^9^ Institute of Fruit Tree and Tea Science, National Agriculture and Food Research Organization (NARO), Morioka, Japan; ^10^ Research Division Plant Breeding, Agroscope, Waedenswil, Switzerland

**Keywords:** apple blotch, *D. coronariae*, *Malus*, genome sequence, short reads, long reads, mating types

## Abstract

*Diplocarpon coronariae* is a fungal pathogen that is prevalent in low-input apple production. Over the past 15 years, it has become increasingly distributed in Europe. However, comprehensive insights into its biology and pathogenicity remain limited. One particular aspect is the rarity of the sexual morph of this pathogen, a phenomenon hitherto unobserved in Europe. *Diplocarpon coronariae* reproduces through a heterothallic mating system requiring at least two different mating types for sexual reproduction. Genes determining the mating types are located on the mating-type locus. In this study, *D. coronariae* strain DC1_JKI from Dresden, Germany, was sequenced and used to unravel the structure of the mating type locus. Using short-read and long-read sequencing methods, the first gapless and near-complete telomere-to-telomere genome assembly of *D. coronariae* was achieved. The assembled genome spans 51.2 Mbp and comprises 21 chromosome-scale contigs of high completeness. The generated genome sequence was used to *in silico* elucidate the structure of the mating-type locus, identified as MAT1-2. Furthermore, an examination of MAT1-1 and MAT1-2 frequency across a diverse set of samples sourced from Europe and Asia revealed the exclusive presence of MAT1-2 in European samples, whereas both MAT loci were present in Asian counterparts. Our findings suggest an explanation for the absence of the sexual morph, potentially linked to the absence of the second mating idiomorph of *D. coronariae* in European apple orchards.

## 1 Introduction

*Diplocarpon coronariae* (Ellis & Davis) Wöhner & Rossman (Crous et al., 2020) is the fungal pathogen causing apple blotch disease on leaves and fruits. The initial description from North America (Davis, 1903) identified the asexual morph, which was designated as *Ascochyta coronaria* and later as *Marssonina coronariae* (Ellis & Davis) Davis. The sexual morph of the fungus has been described as *Diplocarpon mali* (Harada et al., 1974). Presently, the pathogen has a global distribution, with reported high yield losses in Asian apple-growing regions (Shou et al., 2009; Yin et al., 2013; Lian et al., 2021; Park et al., 2023) and low-input apple cultivation in Europe (Lindner, 2012; Naef et al., 2013; Wöhner and Emeriewen, 2019). In Europe, estimating the pathogen’s rate of spread and its ability to adapt to changing climatic conditions remains challenging due to limited studies about the fungus. Crucial drivers for fungal adaptation include rapid generation times, spore production, stress tolerance, genetic diversity within a population, mutation, and the ability for sexual reproduction (Anderson et al., 1992; Leducq, 2014; Zeyl, 2009; Drenth et al., 2019; McDonald and Linde, 2002). Fungi in the *Ascomycota* phylum display various reproductive strategies to ensure their survival and spread (Nelson, 1996; Bennett and Turgeon, 2016; Wilson et al., 2021). The main strategies in these fungi include asexual reproduction for large dispersion during the growing season of the host, whereas sexual ascospores are produced in autumn (Harada et al., 1974). Sexual reproduction is based on either homothallism or heterothallism (Butler, 2007; Naranjo-Ortiz and Gabaldón, 2020). Homothallic organisms are self-fertile and can have either A-type homothallic or A/a-type homothallic characteristics (Pöggeler, 1999; Wilson et al., 2021). A-type homothallic organisms contain only one mating type idiomorph, e.g., MAT1, whereas A/a-type homothallic species contain mating type sequences similar to both A and a idiomorphs (e.g., MAT1 and MAT2). Both are required for self-mating (Pöggeler, 1999). In contrast, heterothallism requires two different individuals and each with distinct idiomorphs (MAT1-1 and MAT1-2) for sexual reproduction (Butler, 2007; Bock et al., 2021).

Genome sequencing has revealed *D. coronariae*’s heterothallic mating system (Cheng et al., 2021). However, a general low frequency of the sexual morph raises questions about the species adaptation to changing environmental conditions. In recent studies, the evolutionary potential of the pathogen *D. coronariae* has been explored extensively. Oberhänsli et al. (2021) hypothesized that *D. coronariae* was recently introduced in Europe and investigated 31 distinct multilocus genotypes within a set of 313 European samples. Although the presence of sexual reproduction has not yet been confirmed in Europe, the study underscores the substantial evolutionary potential of the pathogen. Further supporting this, Cheng et al. (2021) confirmed the genetic structures indicative of a heterothallic mating system for *D. coronariae*, providing evidence for two idiomorphs, MAT1-1 and MAT1-2. Yet, the lack of a chromosome-scale genome sequence for the idiomorph MAT1-2 limits a comprehensive understanding.

The aims of this study were 1) to assemble a genome sequence from the European *D. coronariae* isolate; 2) to identify differences in the genetic structure of the MAT locus of DC1_JKI and NL1; and 3) finally to investigate the occurrence of mating types MAT1-1 and MAT1-2 mating types in samples from Europe and Asia in order to assess whether sexual recombination via heterothallism is possible in Europe.

## 2 Materials and methods

### 2.1 *Diplocarpon coronariae* samples

Various starting material were available for genome sequencing and detection of the mating types. Single spores were isolated from *D. coronariae*-infected leaves from the experimental orchard in Dresden Pillnitz, Germany (51°00′00.7″N 13°53′05.2″E) for subsequent DNA extractions, which were used for sequencing. Acervuli present on the leaves were soaked in sterile water for 30 s and gently scraped with a pipette tip to release the spores. The spore solution was then plated on 1.5% water agar (Otto Nordwald, Germany) supplemented with 25 μg ml^−1^ each of chloramphenicol (Duchefa, Netherlands) and tetracycline hydrochloride (Sigma, USA). After incubation for 18 h to 24 h at room temperature, germinated spores were transferred using a needle under a stereomicroscope (Zeiss, Germany) to potato–carrot dextrose agar (PCDA) supplemented with peptone (Roth, Germany; Zhao et al., 2010). The same antibiotics were added to the PCDA as described above. Plates were sealed with parafilm and cultured at 22°C and diffused white light from in a climate chamber (Percival^®^, model CU-22L). The fungal isolate DC1_JKI was cryo-conserved in saccharose–peptone solution (14% m/v saccharose (Merck, Germany), 1% m/v peptone (Roth, Germany)) at −80°C. For recultivation, the isolate was thawed and suspended in sterile water. A total of 100 µl suspension was spread on agar plates using a Drigalski spatula (Roth, Germany). The Japanese reference isolate of *D. coronariae* (NBRC 30405) was obtained from the National Biology Resource Center (Shibuya-ku, Tokyo, Japan, http://www.nite.go.jp) and cultivated, as described above.

Mating-type determination was conducted on available DNA extracts from the former study of Oberhänsli et al. (2021), which comprised 32 samples of 17 multilocus-type genotypes of *D. coronariae*. These extracts were obtained directly from collected (2016 and 2017) and infected leaves or from isolates grown on cultivation media (Oberhänsli et al., 2021). The samples were collected in Germany, Switzerland, and Italy, and one was obtained from Korea. Further 24 samples from Korea, which were not evaluated in Oberhänsli et al. (2021) but collected within the former project, were available and included in this study. However, the Japanese reference isolate was obtained from the National Biology Resource Center as mentioned before. Additional extracts were tested from samples, which were collected as infected leaf material in 2022 and 2023. Of these samples, 10 originated from Switzerland, 1 from Liechtenstein, 1 from France, 5 from Germany, 10 from Japan, and 30 from Canada. **Table 1** and **Supplementary Table S1** provide summarized and specific information about the samples from this study.

**Table 1:** Samples for sequencing and mating type analysis used in this study.

Origin	Total number	Sample	Provider	Testing facility
**Germany**	11	DE17, DE19, DE63, DE65, DE67, DE14, DE18, DE20, DE21, DE62, DE66	Oberhänsli, T.; FIBL (Oberhänsli et al., 2021)	JKI
	6	DC1_JKI*, CH22-12, CH22-14, RB-01, BY, DD	Patocchi, A.; Agroscope Wöhner, T.; JKI	JKI
**Switzerland**	17	CH001, CH002, CH018, CH019, CH022, CH024, CH025, CH032*, CH033, CH040*, CH042, CH043, CH050, CH052, CH053, CH054, CH068*	Patocchi, A.; Agroscope	JKI
	10	CH22-1, CH22-2, CH22-3, CH22-4, CH22-5, CH22-7, CH22-8, CH22-10,		JKI
**Italy**	2	IT01, IT02	Oberhänsli, T.; FIBL (Oberhänsli et al., 2021)	JKI
**Liechtenstein**	1	CH22-9	Patocchi, A.; Agroscope	JKI
**France**	1	CH22-13	Patocchi, A.; Agroscope	JKI
**Japan**	1	NBRC30405*	Oberhänsli, T.; FIBL (Harada et al., 1974)	JKI
	10	JP22-1–JP22-10	Moriya, S.; NARO	NARO
**Korea**	1	KR01	Oberhänsli, T.; FIBL (Oberhänsli et al., 2021)	JKI
	24	KR06, KR07, KR08, KR09, KR10, KR11, KR28, KR29, KR30, KR48, KR49, KR50, KR71, KR72, KR73, KR74, KR75, KR76, KR77, KR78,	Yeon, I., Oberhänsli, T.; FIBL	JKI
**Canada**	30	CAN1-CAN30	Oberhänsli, T.; FIBL	FIBL

Key details of the samples were described, including their origin, type, total number in this country, provider, and mating type investigator. In bold are the sample accessions, which have been sequenced in this study.

*Sample accessions originally isolated as single spore isolates. JKI, Julius Kuhn Institute; FIBL, Forschungsinstitut für biologischen Landbau; NARO, National Agriculture and Food Research Organization.

### 2.2 DNA extraction

After 21 days of cultivation, 100 mg of fungal mycelium (DC1_JKI) was scraped from the medium and ground to powder a using mortar and pestle under liquid nitrogen. The powder was dissolved in 800 µl BashingBead™ buffer (Quick-DNA Fungal/Bacterial Miniprep Kit, Zymo Research Corp, USA) and transferred to a 2-ml reaction tube. This was followed by centrifugation at 10,000×g for 1 min. All subsequent steps were performed according to the manufacturer’s protocol.

After isolation, the DNA was quantified using a Qubit fluorometer (Thermo Fisher Scientific, USA) and applied to 1% TAE gel for quality and integrity assessment.

For nanopore sequencing, the fungal isolate DC1_JKI and NBRC 30405 was grown in liquid culture for 7 days (potato dextrose broth (Roth, Germany) + 1 m/v % peptone (Fluka, Germany) with 10 vol % vegetable juice (Continental Foods, Belgium); 180 rpm 20°C). Genomic DNA was prepared from 45 mg lyophilized mycelia according to Cenis (1992) with some modifications to avoid disruption of high molecular weight fragments. Briefly, the mycelium was first ground dry to a powder in 2-ml screw cap tubes containing three 3.0-mm ceramic beads 2× for 10 s in a Mixer Mill MM 400 (Retsch, Germany) at a frequency of 30 s^−1^, before adding the extraction buffer containing Proteinase K (Analytik Jena, Germany) and RNAse A (Roth, Germany), both at a final concentration of 0.1 mg/ml. Furthermore, protein precipitation with potassium acetate was repeated twice. DNA was precipitated with addition of the same volume of isopropanol and collected with glass hooks. The glass hooks were dipped in 70% ethanol and air-dried, and DNA was resolved in 200 µl water by incubating at 65°C for 10 min in a heating block. Samples were centrifuged for 10 min at 13,000 rpm (Eppendorf 5430R, Germany), and 0.95 volume of the supernatant was transferred and used for sequencing.

### 2.3 Whole-genome sequencing

A total of 100 ng of high-quality gDNA was sent to Eurofins Genomics (Ebersberg, Germany) for Illumina sequencing (2× 150-bp paired-end read mode). MinION sequencing libraries were prepared according to the Nanopore Sequencing Kit SQK-LSK 109 (Oxford Nanopore Technologies, United Kingdom). Consumables were purchased from New England Biolabs (United Kingdom). A total of 9.8 µg DNA extract was used as input. The kit’s instructions were followed except that the incubation steps during DNA repair and end-prep were extended to 30 min. Short Fragment Buffer was used for washing of the beads. A Flow Cell (R9.4.1) was placed in the MinION Mk1C, prepared following the manufacturer’s instructions (Kit EXP-FLP002), and loaded once with the library. Read event data during the 24-h run generated by MinKNOW software (version 21.11.7) were base-called using Guppy (version 5.1.13) with a quality score cutoff = 15.

The platform Nanopore Galaxy (The Galaxy Community, 2022) was used for processing of the derived reads. Adapter sequences were removed with Porechop (version 0.2.4, Wick, 2017) and *de novo* assembled with Canu (Galaxy Version 2.1.1, estimated genome size 40 m, technology setting for Nanopore, minimum read length 1,000; minimum overlap length 500, target coverage for corrected reads 40, Koren et al. 2017). The Illumina short reads were trimmed with Trim Galore (version 0.6.3, Krueger, 2021) and mapped to the resulting contigs using *bwa-mem (Galaxy Version 0.7.17.4)*. The alignment bam-file was used to polish the genome assembly with *HyPo* (Galaxy Version 1.0.3, mean coverage 90 | approximate genome size 50 m, Kundu et al. 2019).

### 2.4 Quality and completeness assessment of the processed genome data

The NCBI datasets from *Marssonina coronariae* (strain YL1, GCA_037039515.1, ASM3703951v1; strain NL1 GCA_002204255.1, ASM220425v1), *Diplocarpon mali* (strain PGHB, GenBank: GCA_024741835.1, ASM2474183v1), *Diplocarpon rosae* (strain, R4, GenBank: GCA_032760415.1, ASM3276041v1), *Drepanopeziza brunneae* (strain Mb_m1, GenBank: GCA_000298775.1, ASM29877v1), *Blumeriella jaapii* (strain 11BO-GW45, GenBank: GCA_009599575.1, MSU_Bj_1.1), *Monilinia laxa* (strain Mlax316, GenBank: GCA_009299455.1, ASM929945v1), and *Botrytis cinerea* (strain B05.10, RefSeq: GCF_000143535.2, ASM14353v4) were employed for comparative analyses. Quality and completeness of the assembly and annotation were assessed first through BUSCO (Simão et al., 2015) on the Galaxy Europe server (The Galaxy Community, 2022, version 5.4.6 | fungi_odb10 lineage | 549 genomes | 758 BUSCOs). Assembly metrics were obtained using gfastats (Galaxy Version 1.3.6+galaxy0), and the long terminal repeat (LTR) assembly index (LAI) was calculated to assess assembly continuity with comparative datasets (Ou et al., 2018). RepeatMasker (Galaxy Version 4.1.5+galaxy0) was used for the generation of repetitive sequence statistics. The genome size estimation was conducted using k-mer analysis (k: 17) with Illumina short reads from DC1_JKI and NBRC 30405. The Galaxy Server Europe tools Meryl (genomic k-mer counter and sequence utility; Galaxy Version 1.3+galaxy6) and GenomeScope (reference-free genome profiling; Galaxy Version 2.0+galaxy2, Vurture et al. 2017) were utilized. The TTAGGG and CCCTAA telomeric patterns were identified using an R-script (RStudio Server 2022.07.1) with Bioconductor (Biostrings and Biomanager packages), evaluating telomere-to-telomere sequences in windows of 10 kbp for each contig. GC content analysis was performed using isochore (Galaxy Version 5.0.0.1) on the Galaxy Server Australia (Rice et al., 2000; Blankenberg et al., 2007). Circos (Galaxy Version 0.69.8+galaxy7, Krzywinski et al. 2009) was utilized to plot frequencies of genes, repetitive elements, telomere repeats, and GC content. For structural comparisons between genome assemblies, synteny plots were generated using the online tool SynMap2 (Haug-Baltzell et al., 2017) available at the CoGe platform (https://genomevolution.org/coge/). Sequence and annotation data from the comparative datasets were used as input for this analysis. Nucleotide coverage was assessed by mapping (minimap2, Galaxy Version 2.28+galaxy0, preset option up to 5× and 20× sequence divergence) comparative datasets on the reference genome DC1_JKI with subsequent coverage determination using bedtools Genome Coverage (Galaxy Version 2.31.1, Quinlan and Hall, 2010). The mean coverages were plotted as heatmap using the online tool Morpheus (https://software.broadinstitute.org/morpheus) in 100 kilo base windows.

### 2.5 Structural and functional annotation

RepeatModeler2 (version open-1.0.11) was utilized to generate a species-specific repeat library. This process involved the integration of several dependencies, including Tandem Repeats Finder (TRF) v4.09 (Benson, 1999), RECON v1.08 (Bao and Eddy, 2002), RepeatScout v1.0.5 (Price et al., 2005), and RepeatMasker v4.1.4 (Chen, 2004). The configuration was set to use the NCBI search engine. Additionally, long terminal repeat (LTR) structural analysis was enabled, employing GenomeTools v1.6.0 (Gremme et al., 2013), LTR_Retriever v2.9.0 (Ou and Jiang, 2018), Ninja v0.97-cluster_only (Wheeler, 2009), MAFFT v7.471 (Katoh and Standley, 2013), and CD-HIT v4.8.1 (Fu et al., 2012). RepeatMasker version open-4.0.7, using the NCBI/RMBLAST search engine v2.2.27+, was then employed for genome repeat-masking.

Spliced mapping of RNA-seq libraries of other species (**Supplementary Table S2**) was performed with hisat2 version 2.2.1 (Kim et al., 2019) and subsequently merged and sorted with samtools v1.1.3 (Li et al., 2009) using htslib v1.1.3-ds (Bonfield et al., 2021).

BRAKER1 version 3.0.2 (Hoff et al., 2016, 2019) was used to produce an intermediate gene set using the transcriptome evidence by first executing GeneMark-ES Suite v. 4.72 (Lomsadze et al., 2014), removing redundancy in the resulting gene set with DIAMOND v2.0.15.153 (Buchfink et al., 2015), and then training and executing AUGUSTUS v3.4.0 (Stanke et al., 2008). A second intermediate gene set was generated with BRAKER2 (Bruna et al., 2021) v3.0.2, which first executed GeneMark-ET (also from GeneMark-ES Suite v. 4.72, Bruna et al., 2020), which calls GeneMark-ES (Lomsadze et al., 2005), DIAMOND, and Spaln2 (Iwata and Gotoh, 2012) for protein to genome spliced alignment. We use OrthoDB v.11 (Kuznetsov et al., 2023) Fungi partition as protein input (available at https://bioinf.uni-greifswald.de/hubs/dcor/hub.txt). Subsequently, BRAKER2 executed AUGUSTUS. A gene set was generated with TSEBRA (v1.1.2), combining the output of BRAKER1 and BRAKER2 with their respective evidence files (Gabriel et al., 2021). Functional annotation of predicted proteins was performed with InterProScan v5.61-93.0 (Jones et al., 2014). A track hub for displaying the genome via the UCSC Genome Browser (Raney et al., 2024) was generated by MakeHub v1.0.8 (Hoff, 2019). Homology-based gene prediction was used on the target genome assembly (DC1-JKI) by running GeMoMa Pipeline (version 1.9) without gene renaming and synteny check (Keilwagen et al., 2016, 2018). The genome assembly and gene annotation of the following species were used as references in GeMoMa: *Diplocarpon rosae* strain R4 (GenBank: GCA_032760415.1), *Drepanopeziza brunnea* f. sp. ‘multigermtubi’ strain MB_m1 (GenBank: GCF_000298775.1), *Diplocarpon mali* strain PGHB (GenBank: GCA_024741835.1), *Monilinia laxa* strain Mlax316 (GenBank: GCA_009299455.1), and *Botrytis cinerea* strain B05.10 (GenBank: GCF_000143535.2). In addition, the Tsebra annotation was used as external annotation within the GeMoMaPipline.

### 2.6 Phylogenetic analysis

The sequences of 61 universal fungal core genes (Kim et al., 2023) were extracted from seven genome assemblies of the *Dermateaceae* (*Drepanopeziza brunnea* MB_m1*—*GCA_000298775.1, *Diplocarpon rosae* R4—GCA_032760415.1, *Diplocarpon coronariae* DC1_JKI—GCA_964058965.1, *D. coronariae* NBRC 30405—GCA_964059205.1, *D. coronariae* YL1—GCA_037039515.1, *D. coronariae* PGHB—GCA_024741835.1, *D. coronariae* NL1—GCA_002204255.1). The core genes from 51 species of the order Helotiales and the extracted genes were aligned using the UFCG pipeline profile module. The concatenated sequences were then used for phylogenetic tree construction. The evolutionary history was inferred using the Neighbor-Joining method (Saitou and Nei, 1987). The bootstrap tree inferred from 1,000 replicates (Felsenstein, 1985) is taken to represent the evolutionary history of the taxa analyzed. The evolutionary distances were computed using the p-distance method of Nei and Kumar (2000) and are in the units of the number of amino acid differences per site. This analysis involved 65 amino acid sequences. All positions containing gaps and missing data were eliminated (complete deletion option). There were a total of 1,172 positions in the final dataset. Evolutionary analyses were conducted in MEGA11 Tamura et al. (2021). The tree was created with iTOL (Letunic and Bork, 2024).

### 2.7 Mating type detection and *in silico* characterization of MAT locus

DNA extraction of infected leaf material the DNeasy Plant Mini Kit (Qiagen, Germany) and PCR analysis were described in supplements 2.7. and **Supplementary Table S3**. An internal transcribed spacer (ITS) region primer pair (Mc_forward and Mc_reverse; Oberhänsli et al., 2014) was used to confirm the presence of *D. coronariae* on the collected leaf material, following the PCR conditions described in **Supplementary Material 2.7**. The detection of the mating types was performed with two specific primer pairs (MAT1-1-specific_forward, MAT1-1-specific_reverse; MAT1-2-specific_forward, MAT1-2-specific_reverse) for the locus according to Cheng et al. (2021), **Supplementary Material 2.7**. Before PCR, mating-type primers were validated using the BLASTN 2.12.0 (Altschul et al., 1997) algorithm in the CLC Main Workbench (Version 23.0.1) on the sequences DC1_JKI and NBRC 30405.

For *in silico* characterization of the MAT locus in DC_JK1 and NBRC 30405, a BLAST database using the genomic regions of MAT1-2 obtained from *D. coronariae* strain PGHB (GenBank: GCA_024741835.1, ASM2474183v1) and MAT1-1, obtained from *D. coronariae* strain NL1 (GenBank: GCA_002204255.1, ASM220425v1), was generated. Subsequently, we employed this database for the identification of proteins within the MAT locus from both MAT types of DC_JKI and NBRC 30405. Amino acid sequences from MAT1-1 and MAT1-2 were aligned using Miniprot (Galaxy Version 0.12+galaxy0, Li, 2023), providing a detailed overview of the conserved regions and variations within the MAT locus. The resulting alignment was plotted and visualized utilizing the ggplot2 package (Version 3.4.2; Wickham, 2016). For the identification of variants, gaps, and deletions a global alignment using the NCBI Needleman-Wunsch algorithm was performed with CLC Main Workbench (Version 21.0.5).

## 3 Results

### 3.1 Genome sequencing and assembly

**Figure 1:**
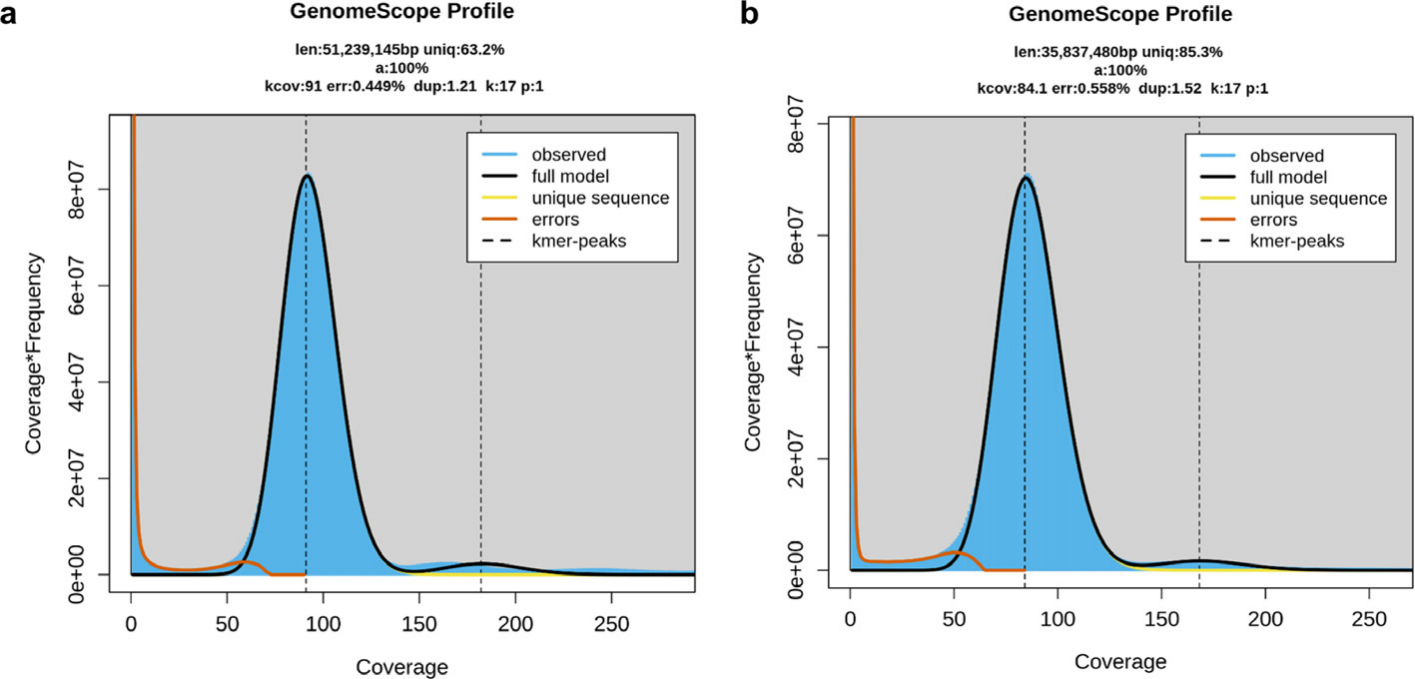
GenomeScope (Galaxy Version 2.0) was utilized to estimate the genome size of *D. coronariae* **(A)** DC1_JKI and **(B)** NBRC 30405, leveraging k-mer counts obtained through Meryl software (Galaxy Version 1.3+galaxy2). Both tools are seamlessly integrated on the GalaxyServerEurope platform. The analysis revealed k-mer peaks corresponding to 17-base pair sequences occurring in a homozygous state (90× depth) within the genome. The coverage depth of individual k-mers was assigned as the respective coverage.

The paired-end Illumina sequencing of the European isolate DC1_JKI, generated a total of 6.3 Mbp from 21 million reads. The k-mer analysis (**Figure 1A**) estimated the haploid genome size to be approximately 51.2 Mbp with k=17. Therefore an 87× median coverage of the estimated genome size was achieved. The same isolate was further sequenced utilizing a MinION R9.4.1 flow cell, yielding 5.34 million raw reads (9.25 G-bases) over a 22-h run time. Following quality filtering (min_qscore= 7) and adapter trimming, 4.68 million reads comprised a total of 8.24 G bases, representing a ~165× coverage of the estimated haploid genome size. After polishing, the final genome assembly, was scaffolded into 21 gapless contigs with an N50 of 4 Mbp. Additionally, a mitochondrial sequence of 77,646 bp was identified. The telomere analysis revealed a total of nine telomere-to-telomere, five partial telomeric, and seven sequences without telomeric regions (**Supplementary Figure S1**).

For the reference isolate NBRC 30405, the paired-end Illumina sequencing data generated a total of 3.32 Gb. The k-mer analysis (**Figure 1B**) estimated the genome size to be ~35.8 Mbp with k=17. Using a MinION R10.4.1 flow cell, we achieved ~84.1× coverage of the estimated genome size of 50.7 Mbp. After polishing, the final assembly, designated as NBRC 30405, consisted of 742 gapless contigs with an N50 of 0.3 Mbp.

### 3.2 Genome annotation and characteristics

The BUSCO analysis indicated a high level of genome completeness for both assemblies, with DC1_JKI at 98.5% and NBRC 30405 at 97.4%, comparable with other fungal genomes (**Table 2**). The LAI was found to be higher for NBRC 30405 compared with DC1_JKI. Analyzing repetitive elements in DC1_JKI, a total of 28,420 elements were identified, constituting 34.15% of the genome. These elements included 20.74% LTR/Copia, 5.67% Ty3-retrotransposons, 0.29% DNA transposons, and 1.14% simple repeats. For NBRC 30405, a total of 34.59% (27,802 elements) were detected, including 20.45% LTR/Copia, 6.20% Ty3-retrotransposons, 0.29% DNA transposons, and 1.64% simple repeats. For gene annotation, BUSCO analysis also shows a high degree of completeness for both assemblies with 99.8% for DC1_JKI and 99.5% for NBRC 30405 (**Table 3**). DC1_JKI harbored a total of 10,940 genes, along with 12,302 mRNAs. NBRC 30405, on the other hand, had 10,816 genes and 12,229 mRNAs annotated.

**Table 2:** BUSCO and assembly statistics of the genomic data generated in this study and comparative datasets (n: 758).

Fungal species		*Diplocarpon coronariae*	*Diplocarpon coronariae*	*Diplocarpon mali*	*Diplocarpon rosae*	*Drepanopeziza brunnea*	*Blumeriella jaapii*	*Monilinia laxa*	*Botrytis cinerea*
Assembly		*DC1_JKI*	*NBRC 30405*	*ASM2474183v1*	*ASM3276041v1*	*ASM29877v1*	*MSU_Bj_1.1*	*ASM929945v1*	*ASM14353v4*
Strain		DC1_JKI	NBRC 30405	PGHB	R4	MB_m1	11BO-GW45	Mlax316	B05.10
BUSCO (%)	C:	98.5	97.4	98.3	98.7	98.9	96.3	98.3	99.2
	S:	98.5	97.0	98.3	98.7	98.8	96.2	98.3	99.2
	D:	0.0	0.4	0.0	0.0	0.1	0.1	0.0	0.0
	F:	0.7	1.3	0.8	0.5	0.5	1.6	0.9	0.5
	M:	0.8	1.3	0.9	0.8	0.6	2.1	0.8	0.3
Assembly statistics	Number of scaffolds	22	742	979	16	89	95	49	18
	Number of contigs	22	742	2149	16	2415	95	49	18
	Total length (Mbp)	51.5	50.7	51.7	36.9	51.9	47.4	42.8	42.6
	Percent gaps	0.0	0.0	0.13	0.0	0.45	0.0	0.0	0.0
	Scaffold N50 (Mbp)	4	0.3	0.12	2	1	1	2	2
	Contigs N50 (Mbp)	4	0.3	0.05	2	0.04	1	2	2
	LAI (GW)	1.4	2.3	0	3.3	6.6	6.9	–	–

C, complete busco; S, single busco; D, duplicated busco; F, fragmented busco; M, missing busco; LAI (GW), genome wide long terminal repeat assembly index

**Table 3:** Key statistics of RepeatMasker analysis on repetitive sequence analysis on long- and short-read (DC1_JKI, YL1, NBRC 30405) and short-read (NL1, PGHB) assemblies from *Diplocarpon coronariae*.

Category	DC1_JKI	YL1	NBRC 30405	NL1	PGHB
Total length	51,493,264 bp	54,484,178 bp	50,384,226 bp	50,267,101 bp	50,267,101 bp
GC content	44.39%	44.08%	45.09%	44.54%	44.85%
Bases masked	17,584,167 bp (34.15%)	19,730,873 bp (36.21%)	16,872,244 bp (33.49%)	18,721,810 bp (37.28%)	15,837,611 bp (31.51%)
Retroelements	8,361 (30.17%)	9,413 (32.14%)	13,098 (29.57%)	8,017 (31.91%)	9,890 (30.47%)
LINEs	116 (0.37%)	87 (0.31%)	203 (0.36%)	107 (0.42%)	113 (0.39%)
LTR elements	8,245 (29.80%)	9,326 (31.83%)	12,895 (29.22%)	6,275 (24.96%)	8,668 (27.25%)
DNA transposons	490 (0.29%)	580 (0.32%)	500 (0.29%)	330 (0.36%)	410 (0.35%)
Unclassified	5,675 (2.31%)	5,816 (2.27%)	5,526 (2.34%)	3,492 (2.58%)	4,722 (2.53%)
Small RNA	20 (0.06%)	47 (0.24%)	18 (0.08%)	21 (0.07%)	30 (0.08%)
Simple repeats	12,383 (1.14%)	12,372 (1.08%)	11,074 (1.05%)	8,572 (1.08%)	10,690 (1.12%)
Low complexity	1,504 (0.17%)	1,488 (0.16%)	1,364 (0.15%)	1,580 (0.16%)	1,300 (0.14%)

### 3.3 Inter- and intraspecific genome sequence comparison

**Figure 2:**
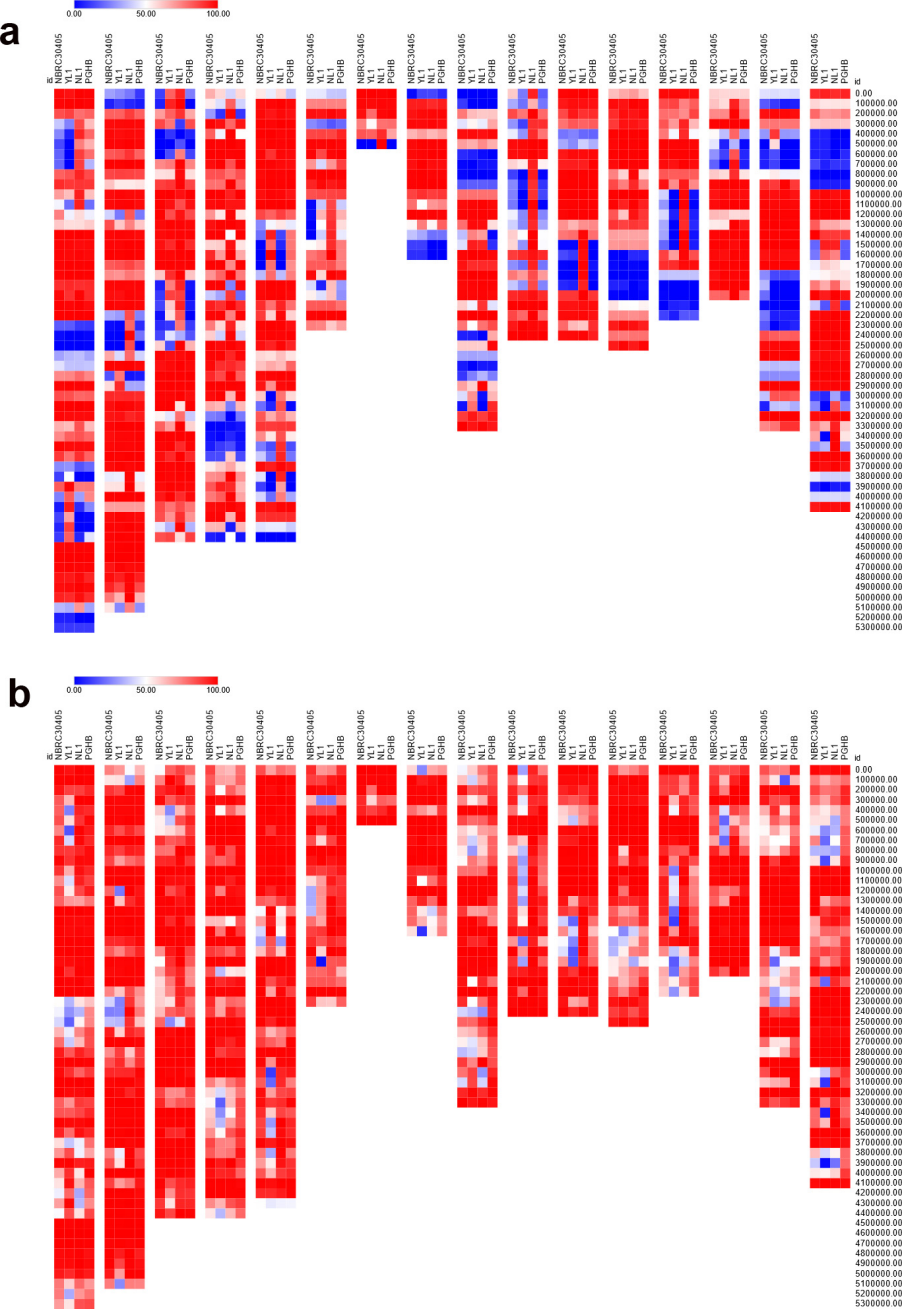
Heatmap of nucleotide coverage after mapping of long- and short-read (NBRC 30405, YL1) and short-read (NL1, PGHB) assemblies to the long- and short-read assemblies of DC1_JKI. The coverage was plotted from 0% (blue) to 50% (white) to 100% (red). **(A)** Only sequences with a stringency of up to <5% sequence difference were mapped. **(B)** Only sequences with a stringency of up to <20% sequence differences were mapped. The sequences with a length smaller than 100 kilo base pair (tig00000004, tig00000007, tig00000021, tig00000022) were not plotted.

In this study, we analyzed the intraspecific nucleotide coverage of *D. coronariae* by comparing short-read assemblies from strains PGHB and NL1 with combined long- and short-read assemblies from strains NBRC 30405 and YL1, using the European strain DC1_JKI as the reference genome. The analysis was performed at two levels of mapping stringency: up to 5% and up to 20% sequence difference. At a mapping stringency of up to 5%, the nucleotide coverages were as follows: NBRC 30405 achieved a coverage of 66.72%, YL1 had a coverage of 66.98%, NL1 exhibited a higher coverage at 76.84%, and PGHB had the lowest coverage at 65.86%. These results indicate that NL1 displayed the most comprehensive alignment to the reference genome under stringent conditions, whereas PGHB showed the least coverage. When the stringency was relaxed to allow up to 20% sequence difference, the nucleotide coverages significantly increased across all strains. NBRC 30405 showed a coverage of 85.95%, YL1’s coverage increased to 76.40%, NL1 further improved to 87.36%, and PGHB achieved the highest coverage at 91.20%. **Figures 2A, B** depict the coverage variation in genomic regions within 100-kbp windows using DC1_JKI as reference.

We further compared the repetitive regions detected by RepeatMasker between short-read assemblies (PGHB and NL1) and hybrid long- and short-read assemblies (NBRC 30405 and YL1) of *D. coronariae*, using the European strain DC1_JKI as the reference genome. Key differences are summarized in **Table 3**. Notably, the hybrid assemblies (NBRC 30405 and YL1) showed a slightly lower percentage of bases masked as repetitive elements compared with the short-read assemblies. For instance, NL1 had 37.28% of its genome masked, the highest among all strains, whereas NBRC 30405 had 33.49% masked. Additionally, the hybrid assemblies contained a higher number of retroelements, with NBRC 30405 detecting the highest number (13,098) compared with the short-read assemblies, which had fewer (8,017 in NL1).

**Figure 3:**
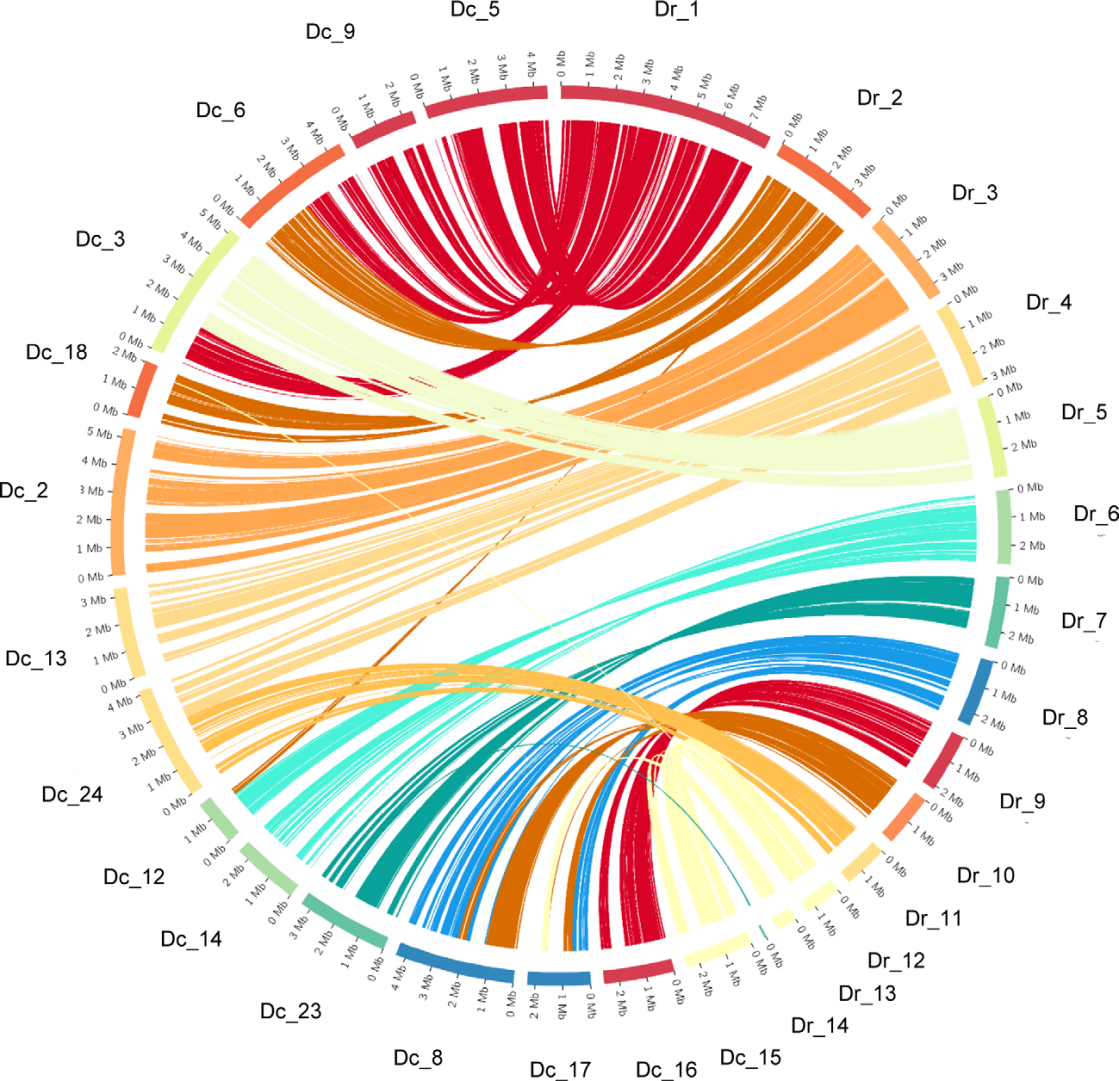
Synteny plot between *D. coronariae* (Dc) and *D. rosae* (Dr) genomes. The circular plot shows the synteny between chromosome-scale sequences of *D. coronariae* on the left site and sequences of *D. rosae* on the right site.

A comparison of the DC1_JKI assembly and annotation with the reference genome of *D. rosae* revealed insights into the genomic architecture and synteny between these two closely related species (see **Figure 3**). We identified 15 chromosome-scale sequences within DC1_JKI that displayed significant synteny with 14 chromosome-scale sequences in the *D. rosae* genome. Further investigation into the syntenic relationships uncovered a total of 65 syntenic blocks (**Supplementary Table S4**), characterized by conserved gene order. Notably, we observed synteny between Dr_3 and Dc_2, Dr_5 and Dc_3, Dr_7 and Dc_23, Dr_9 and Dc_16, Dr_11 and Dc_24, and Dr_12 and Dc_15.

This underscores the broader scope of conserved genomic regions across these specific gene loci. Additionally, Dc_5 and Dc_9 exhibited synteny with Dr_1, whereas Dc_6 exhibited partial synteny. Similar arrangements were observed for Dc_6 and Dc_18 that shared synteny with Dr_2. Others were found between Dr_4 and Dc_13/Dc_24, Dr_6 and Dc_12/Dc_14. Interestingly, Dr_8 and Dr_10 exhibited synteny with both Dc_8 and Dc_17 underlining the complexity of genomic rearrangements and shared gene order between the two species.

### 3.4 Phylogenetic analysis

**Figure 4:**
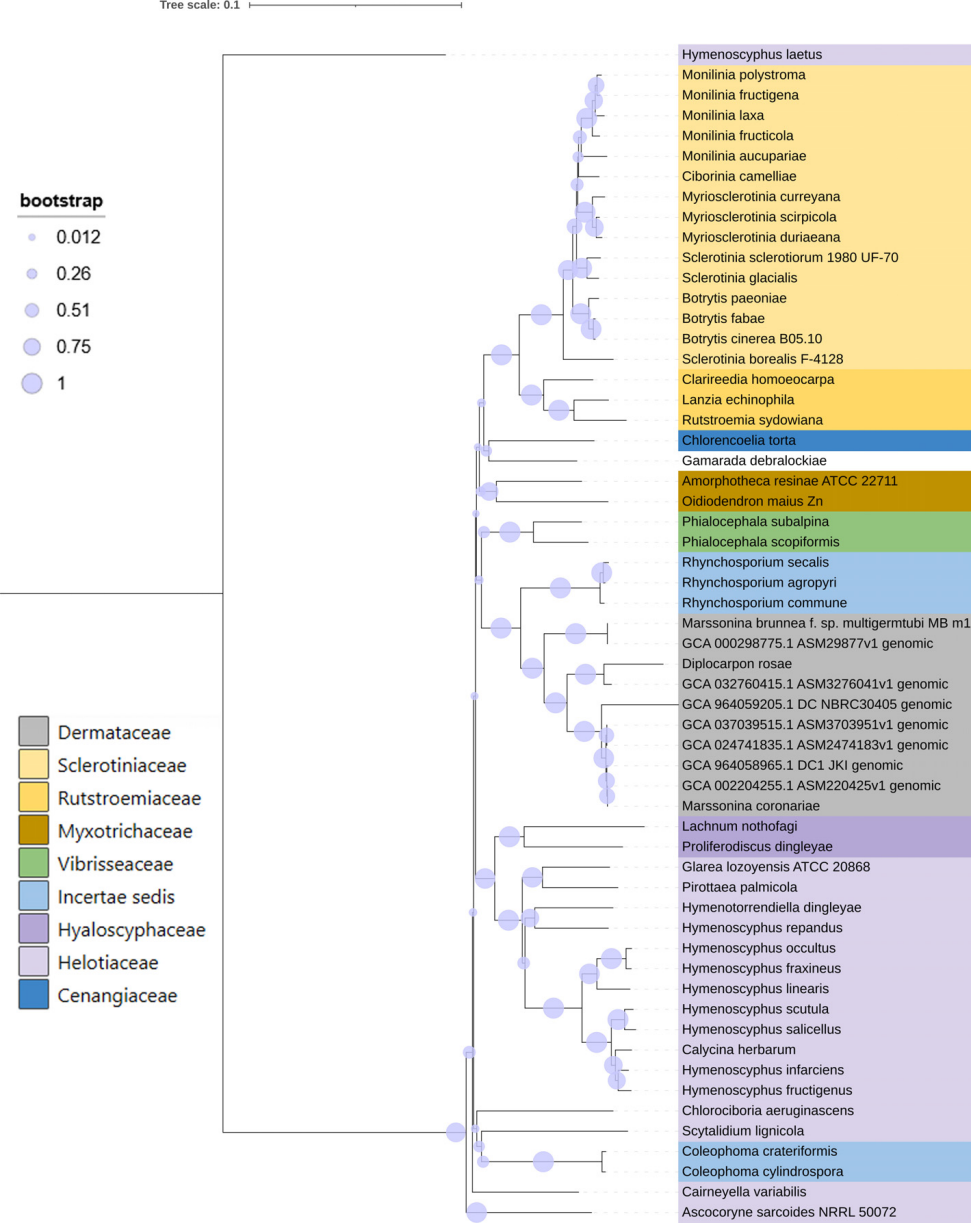
Phylogenetic analysis of fungal species in the order *Helotiales*. The concatenated alignments of 61 UFCG marker sequences from 51 species (Kim et al., 2023) and extracted genes from seven genome assemblies (*D. coronariae* DC1_JKI, NBRC 30405, YL1, NL1, PGHB; *D. rosae* R4; *Drepanopeziza brunnea* Mb_m1) were used for the construction of the tree (Neighbor joining, 1,000 bootstraps) and Kimura 80-nucleotide distance measure.

A phylogenetic tree was constructed using concatenated 61 universal core sequences from fungal species of the family Helotiales, including *Diplocarpon coronariae* DC1_JKI, YL1, NL1, PGHB, NBRC 30405, and *D. rosae* (R4) and *Drepanopeziza brunneae* (MB_m1). The phylogenetic tree (**Figure 4**) revealed distinct relationships among the species. Notably, DC1_JKI and NL1 exhibited a closer phylogenetic relationship compared with *D. mali* (YL1) and PGHB. Furthermore, the *Diplocarpon coronariae* strains DC1_JKI, NBRC 30405, PGHB, NL1, and YL1 formed a cohesive cluster, indicating a close genetic relationship.

### 3.5 MAT locus detection and initial characterization of *Diplocarpon coronariae* samples from Europe, Asia, and Canada

Genomic investigations unveiled distinctive features within the MAT loci across the strains. The MAT1-1 locus in NBRC 30405 was localized precisely to tig00000469 (583,848 bp–597,738 bp) with a length of 13,890 bp, whereas the MAT1-1 sequence in NL1 (MZNU01000336.1 | 398,255 bp–412,134 bp) had a comparable length of 13,879 bp. Similarly, the MAT1-2 locus in DC1_JKI was identified on tig00000009 (937,282 bp–950,551 bp) with a total length of 13,269 bp, whereas the MAT1-2 sequence in PGHB showed a slight variation in length with 13,238 bp.

We identified 107 single-nucleotide polymorphism (SNP) sites between MAT1-2 of DC1_JKI and PGHB as well as 62 SNPs between MAT1-1 of NL1 and NBRC 30405. A total of four deletions were found in PGHB and three in DC1_JKI as well as three in NL1 and five in NBRC 30405.

**Figure 5:**
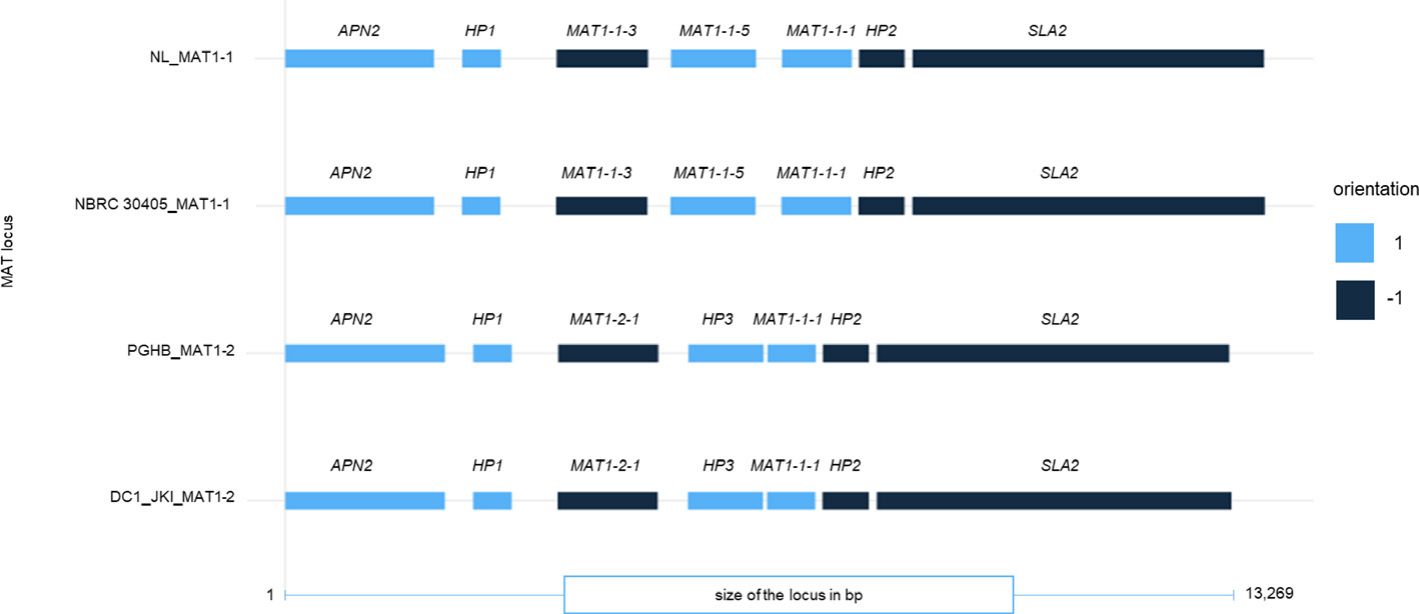
Protein annotation of the typical genes in the MAT1-1 and MAT1-2 locus of *D. coronariae.* The strains NL1 and NBRC 30405 represent the Mating Type MAT1-1, whereas PGHB and DC1_JKI represent the MAT1-2 locus. *APN2* (DNA purinic/apyrimidinic lyase 2), *HP1, HP2, HP3* (hypothetical proteins 1 to 3), *MAT1-1-1* (alpha-box protein), *MAT1-2-1* (high-mobility group (HMG) motif-containing protein), *MAT1-1-3* (transcription factor), *MAT1-1-5* (unknown function), *SLA2* (cytoskeleton assembly control).

Miniprot was used to predict the protein sequences based on the homology of the reference sequence from NL1 and PGHB and locus positions due to Cheng et al. (2021) for each MAT1-1 and MAT1-2 protein of NBRC 30405 and DC1_JKI. When comparing the order of obtained proteins, no large structural differences were observed (**Figure 5**). A global alignment using the NCBI Needleman–Wunsch algorithm resulted in minor differences (identities between 99% and 97%) between the protein sequences of DNA Lyase 2, hypothetical protein 1, MAT1-1-3, MAT1-1-5, hypothetical protein 2, and SLA2. The largest difference was found between MAT1-1-1 of NL1 and NBRC 30405 and MAT1-1-1 of DC1_JKI and strain PGHB. No differences were found between the MAT1-2-1 between DC1_JKI and PGHB.

The primers were validated by a BLAST search against the sequenced genomes, resulting in a complete match of the primers on the sequences shown in **Table 4** and **Supplementary Tables S5**, **S6**. On contig tig000009 (DC1_JKI), both primers for MAT1-2 were found with an E-value of 2.07E−05, but not the complete primer pair for MAT1-1. However, these primers were successfully identified on contig tig00000469 (NBRC 30405) with an E-value of 2.04E−05, although the primer pairs for MAT1-2 were not. No other complete primer sequences were identified in the two genomes.

**Table 4:** Results of the primer validation via BLASTN.

Assembly	Primer	Hit	E-value	Score	HSP start	HSP end	HSP length	%Identity	%Gaps
**DC1_JKI**	MAT-1-2-specific_forward	tig00000009	2.07E−05	46	941186	941208	23	100	0
	MAT-1-2-specific_reverse	tig00000009	2.07E−05	46	941661	941639	23	100	0
**NBRC 30405**	MAT1-1-specific_forward	tig00000469	2.04E−05	46	589665	589687	23	100	0
	MAT1-1-specific_reverse	tig00000469	2.04E−05	46	590132	590110	23	100	0

For each primer, the parameters, hit sequence, E-value, score, HSP (high-scoring segment pair) start and end positions, HSP length, percentage identity, and percentage gap, are given and provide insights into the effectiveness and specificity of the primers.

The initial characterization of the European samples via PCR reveals an exclusive presence of the MAT1-2 mating type (**Table 5**; **Supplementary Table S7**). MAT1-1 was absent in all tested samples from Europe, irrespective of variations in multilocus genotypes within a country or across different European countries. Furthermore, no evidence of MAT1-1 occurrence was found in non-genotyped samples collected in Europe. As in European samples, Canadian samples demonstrated a distinct profile. In 29 samples the presence of MAT1-2 was detected uniquely.

**Table 5:** Samples used for mating type analysis and their assignment for the specific mating type according to Cheng et al. (2021).

Origin	Total number	MAT1-2	MAT1-1	MAT1-1 and MAT1-2
Germany	12	DE14, DE18, DE20, DE21, DE62, DE66, CH22-12, CH22-14, RB-01, BY, DD, DC1_JKI		
Switzerland	26	CH001, CH002, CH018, CH019, CH022, CH024, CH025, CH032, CH033, CH040, CH042, CH043, CH050, CH052, CH053, CH054, CH068, CH22-1, CH22-2, CH22-3, CH22-4, CH22-5, CH22-7, CH22-8, CH22-10, CH22-11		
Italy	2	IT01, IT02		
Liechtenstein	1	CH22-9		
France	1	CH22-13		
Japan	11	JP22-4, JP22-6, JP22-7	JP01 (NBRC 30405), JP22-3	JP22-1, JP22-2, JP22-5, JP22-8, JP22-9, JP22-10
Korea	19	KR48, KR71	KR01, KR06, KR07, KR08, KR10, KR28, KR29, KR49, KR74, KR75, KR77, KR78	KR09, KR11, KR50, KR72, KR73
Canada	29	CAN1–CAN22, CAN24–CAN30		

In contrast, the Asian samples emerged in a diverse pattern. From the Japanese samples, two were tested positive for MAT1-1, three for MAT1-2, and six for both mating types. Similarly, among the 20 Korean samples, 12 were tested uniquely positive for MAT1-1 and two for MAT1-2. Notably, six samples displayed PCR results indicating the presence of both mating types, further highlighting the genetic complexity within Korean samples.

Among the 114 samples, six exhibited no positive PCR amplification for either MAT1-1 or MAT1-2. In the same subset of samples, amplification issues, indicating low DNA amounts and poor sample quality, were observed in *D. coronariae*-specific ITS controls. In addition, seven samples with positive evidence for the specific ITS region but no amplification for either mating type were observed. These samples, as well as one Canadian sample and five Korean samples with the same issues, were excluded from the analysis and not presented in **Table 5** and **Supplementary Table S3**.

## 4 Discussion

A complete or near-complete genome assembly is very useful to advance the understanding of the genetic makeup and evolutionary dynamics of organisms. In the case of *Diplocarpon coronariae*, the existing genomic database at NCBI currently features two assemblies with the old taxonomic nomenclature, specifically strain PGHB *(Diplocarpon mali*, originated from China) and strain NL1 (*Marssonina coronariae*, originated from China). These assemblies are scaffolded but lack chromosomal sequences due to their reliance on short-read technologies. Since 2024, a new hybrid and chromosome scale assembly like DC1_JKI from the Asian strain YL1 is available. In contrast, the genomic sequence of DC1_JKI represents the first gapless-assembled and nearly complete telomere-to-telomere genome sequence of *D. coronariae* from a European isolate. In addition, a genome sequence of the Japanese strain NBRC 30405 (Shibuya-ku, Tokyo, Japan, http://www.nite.go.jp) was assembled to compare with the European strain.

The chromosomal sequence assembly of *D. coronariae*, exemplified by the European isolate DC1_JKI, provided essential genomic information on the composition and structure of this pathogen’s genome, which has also been previously shown for other pathogens such as *Monilinia laxa* (Landi et al., 2020) *Blumeriella jaapii* (Peng et al., 2020), *Diplocarpon rosae* (Neu et al., 2017), and many others (Möller and Stukenbrock, 2017). With an estimated genome size of 51.2 Mbp, the final assembly consisted of 21 gapless contigs and one mitochondrial sequence, exhibiting high completeness and contiguity in comparison with other species from the family Helotiales (**Table 2**). This represents a significant advancement compared with the 979 contigs of strain PGHB and 589 contigs of strain NL1 (Cheng et al., 2021). Our comparative analysis with the reference genome of *D. rosae* revealed 15 chromosome-scale sequences demonstrating significant synteny, indicative of conserved genomic regions, of which nine chromosomes were completely sequenced telomere-to-telomere. Similar results were observed for fungi from the *Magnaporthaceae* (Okagaki et al., 2015), *Cutaneotrichosporon* species (Kobayashi et al., 2023), *Hypocreale* (Saud et al., 2021), or *Colletotrichum* (Rao and Nandineni, 2017). The assembly size is furthermore supported by the second genome size estimation based on k-mers, which resolved the same length of 51.2 Mbp for *D. coronariae* strain DC1_JKI. However, the sequence of the Japanese reference isolate NBRC 30405 resulted in a gapless assembly consisting of 742 contigs with a total length of 50 Mbp. K-mer analysis of this strain resulted in an estimation of 35.8 Mbp, which can be attributed to a lower coverage of input short reads or the large number of repetitive elements (Pflug et al., 2020).

The limitations associated with short-read sequencing technologies hinder the generation of gapless assemblies and telomere-to-telomere sequencing (Petersen et al., 2022). In contrast, long-read sequencing technologies, as employed in this study, offer a distinct advantage by generating longer reads that can cover structurally challenging sequences, including repetitive regions like telomeres (Amarasinghe et al., 2020). This enables the assembly of contiguous sequences without gaps, facilitating the comprehensive mapping of entire chromosomes. In this study, we demonstrated that our hybrid assemblies (NBRC 30405, YL1) achieved a slightly lower percentage of bases masked as repetitive elements compared with short-read assemblies (NL1 and PGHB), yet they uncovered a higher number of retroelements. This is consistent with other studies in *Ascomycetes*, where long-read-based assemblies have shown superior performance in resolving complex genomic regions, including large indels, transposable elements, and other structural variants (Wang et al., 2021; Ogaji et al., 2022). The ability to assemble these difficult regions is crucial for a more accurate and complete representation of the genome, which short-read assemblies often fail to achieve due to their limited read lengths and challenges in spanning repetitive sequences. Moreover, when comparing base coverage of available Asian assemblies to DC1_JKI, we found that the short-read assemblies (NL1 and PGHB) exhibited higher base coverages than the hybrid assemblies (YL1 and NBRC 30405). This finding suggests that while hybrid assemblies may not always achieve higher coverage or completeness in terms of raw base pairs, they provide superior continuity and accuracy in relation to chromosome structure, particularly in regions with high repeat content or structural complexity. This study, therefore, underscores the importance of integrating long-read sequencing technologies to overcome the inherent limitations of short-read assemblies, especially in organisms with complex genomes like *Ascomycetes*. By improving the resolution of repetitive regions and structural variants, hybrid assemblies contribute to a more accurate and functionally relevant genome assembly, which is essential for downstream applications in genomics and evolutionary studies.

This study provides a phylogenetic analysis, to classify the relationship of the European strain DC1_JKI, involving various fungal species. This underscores the expected close relationship between DC1_JKI, NBRC 30405, and *D. coronariae* isolates, emphasizing their shared evolutionary history. Moreover, the close relationship within the genus *Diplocarpon* was also corroborated by *D. rosae*, as previously demonstrated by Cheng et al. (2021). These findings establish a robust foundation for future investigations into the genetics, evolution, and pathogenicity of *D. coronariae* and support that the European and Asian isolates belong to the same species. Discrepancies in the nomenclature of the fungus still persist and scientific publications continue to emerge, which refer to the pathogen by its asexual stage or a synonym. These varying designations may raise suspicion among critical reviewers. This study, based on genomic data, can substantiate that the fungus in Europe belongs to the same species as that found in Asia, which is correctly termed *Diplocarpon coronariae* (Crous et al., 2020).

*Diplocarpon coronariae* is assumed to have a heterothallic mating system, meaning its genotypes carry either the MAT1-1 or MAT1-2 locus (Cheng et al., 2021). For sexual reproduction to occur, individuals with different MAT loci must interact, resulting in the formation of sexual spores and the sexual stage. Interestingly, observations of the sexual stage in *D. coronariae* are infrequent, documented only twice in Asia since the pathogen’s initial description (Harada et al., 1974; Gao et al., 2011; Sutton et al., 2014). Notably, this reproductive stage has not been observed in Europe, where the pathogen was first reported in Italy in 2001 (Tamietti and Matta, 2003). This lack of observation in Europe could be due to the low examination frequency. Another possibility is the recent introduction of the fungus in Europe with only one of the mating types. In the latter case, sexual reproduction is not expected.

Using the genomic information of the current study, we aimed to characterize the MAT locus of European *Diplocarpon coronariae* strain DC1_JKI by comparing its structure to available loci from NCBI datasets and the Japanese strain NBRC 30405. Our findings revealed that the European strain DC1_JKI carries MAT1-2, and the strain NBRC 30405 originated from Japan was found to carry MAT1-1. Therefore, the Japanese strain carry the same mating type as the strain NL1 from China (Cheng et al., 2021). However, in China both mating types were observed and also strains with MAT1-2 are known (YL1, YL5 and YL11; Cheng et al., 2021). We moreover observed a length variation within the locus. Cheng et al. (2021) described the MAT1-1 and MAT1-2 with a genomic range of up to 9,000 bp. The observed loci in DC1_JKI and NBRC 30405 exhibited a length of up to approximately 14,000 bp.

To investigate the distribution of MAT loci across different regions, additional 101 *D. coronariae* samples from Middle Europe and Asia have been examined. In the European samples, only MAT1-2 was found, whereas both mating types were present in Asian samples from Korea and Japan. This led us to speculate that the absence of MAT1-1 in Europe might be the reason for the absence of the sexual stage, although the limited number of European samples included in the study does not allow the definitive exclusion of MAT1-1 in Europe. Oberhänsli et al. (2021) identified 31 multilocus genotypes in 313 samples, and 17 out of the collection were analyzed for their mating type as DNA from the previous study was available yet. As heterothallism of *D. coronariae* in Europe looks to be absent, the genetic diversity observed in this later study is also unlikely to depend on sexual recombination due to heterothallism, as only one mating type (MAT1-2) was observed.

A few leaf samples from Korea and Japan exhibited both mating types. The genome sequences presented in this study (DC1_JKI and NBRC 30405), along with the Chinese isolate NL1 (Cheng et al., 2021), confirm that *Diplocarpon coronariae* is a heterothallic organism, possessing only a single mating type region in each genome. Therefore, homothallism can be excluded as a cause of the presence of both mating types in these samples. A mixture of different strains with distinct mating types is unlikely, and results may be a product from contamination or technical issues with the PCR process. Given that the samples were not isolated from single spores, contamination during or after DNA isolation remains a plausible explanation. To address these concerns, further testing, including the preparation and culture of single-spore isolates, followed by DNA analysis, would provide a more definitive understanding of the findings. Extensive testing of the Asian fungal population is also necessary to clarify these inconclusive results. Until then, the potential for contamination or technical artifacts should be acknowledged as a limitation in this study.

Nevertheless, the two mating types in Asian samples demonstrated that the genetic basis for heterothallism is present, confirming the results from Cheng et al. (2021). Despite this, there is also a lack of observations regarding the sexual form in Asia. In this study, samples were analyzed that were determined to be *D. coronariae* by positive detection of the ITS region but did not show amplification of the mating type. This result could be an indication that there are further types in addition to the already known mating types as known for *Basidiomycetes* (Kuees, 2015). It is also possible that the sequence of the locus was fragmented during isolation, which is more likely. Furthermore, we investigated a small set of samples from Canada, and only MAT1-2 was detected. However, for both investigated regions in Europe and Canada, a large-scale sampling would be necessary to finally clarify the presence or absence of MAT1-1 in Europe and North America. A comparative situation is described for the pathogen *Ascochyta rabiei* in Australia (Mehmood et al., 2017), where also only one mating type was present. The results and insights from these studies could be useful for a larger survey on *Diplocarpon coronariae* in Europe, Asia, and North America. To date, the distribution of the pathogen was described in 17 countries (North America, South America, Asia, and Europe, Wöhner and Emeriewen (2019)).

The absence or the rarity of a second mating type in fungal pathogens typically results in a reduction in the pathogen’s capacity to adapt to novel environments and evolve, although there are other drivers for adaptation (Seidl and Thomma, 2014). The absence of the mating type can impede the development of new virulence traits, which breaks the resistance of cultivated apple, or resistances to control measures. An understanding of the mating system of a pathogen is essential for the development of effective and sustainable disease management strategies, including the creation of resistant crop varieties and the application of fungicides. Furthermore, the presence of a single mating type could limit the geographic spread of a pathogen in comparison with a pathogen with active sexual recombination, which produces a greater diversity of offspring. However, considering the limited sampling scope for mating-type analysis, we advocate for broader investigations across diverse locations in future research projects.

## Data Availability

The data presented in the study are deposited in the ENA repository (www.ebi.ac.uk/ena), accession number GCA_964059205.1 & GCA_964058965.1. Additional data can be found in the OpenAgrar repository (www.openagrar.de, Richter et al. 2024).
