# Supplementary material for: Genome sequence of a European Diplocarpon coronariae strain and in silico structure of the mating-type locus
Source: Front Plant Sci. 2024 Oct 18;15:1437132. doi: 10.3389/fpls.2024.1437132 (PMC11527701; doi:10.3389/fpls.2024.1437132)
Supplement: Supplementary file 1 [file DataSheet1.docx]

Supplementary Material

# Supplementary Methods

Supplements 2.7. DNA Extraction and PCR

Infested apple leaves were either stored frozen at -18 °C or dried and kept at room temperature until DNA extraction. DNA was extracted using the DNeasy Plant Mini Kit (Qiagen, Germany). A total of 20 mg of dry leaf material or 100mg of frozen leaves were ground to powder using a mixer mill MM200 (Retsch, Germany) and proceeded according to the manufacturer’s instructions. DNA was quantified using a Nanodrop OneC spectrophotometer (Thermo Scientific, USA) and diluted to concentration of 20 ng/µl for the polymerase chain reaction (PCR). The PCR was performed in a total volume of 25µl containing 20ng of genomic DNA, 1x DreamTaq Buffer, 10mM dNTPs, 0.4µM of each primer (Supplemental material Table 2), 0.005% BSA, 1% PVP (K30) and 0.5U DreamTaq Polymerase (Thermo Fisher Scientific). The PCR started with an initial denaturation for 3min at 94˚C, followed by 35 cycles of 30s denaturation at 94˚C, 1min annealing at 60˚C and 30s elongation at 72˚C. After the final elongation at 72˚C for 3min, the reactions were cooled down to 10˚C and the PCR products were analysed by gel electrophoresis (2 % agarose supplemented with ethidium-bromide) in 1x Tris-acetate-EDTA buffer. The internal transcribed spacer (ITS) region primer (Oberhänsli et al., 2014) were used to confirm the presence of *Dc* in the isolate. Detection of the mating type in the different strains was performed with two specific primer pairs for the locus according to Cheng et al. 2021.

The analysis of the Canadian, and in parallel that of some European samples was also tested with SYBR quantitative PCR in a total volume of 10 µl. To this end, the extracted genomic DNA added to the qPCRBIO SyGreen Blue Mix ([www.pcrbio.com](http://www.pcrbio.com)) containing 0.3 µM each of forward and reverse primer. The qPCR was performed in a BioRad CFX96 with 3 min of enzyme activation at 95°C followed by 40 cycles with 10 s at 95°C (denaturation) and 40 s at 60°C (annealing/elongation). After each cycle fluorescence was recorded with the FAM/SYBR-Green filter. The dissociation temperature of the PCR products was assessed with a temperature gradient from 65 – 95°C, using 0.5°C increment steps of 10 s each.

Typical qPCR cycle numbers for positive reactions with the MAT1-1 and MAT1-2 were between 20-25 cycles and the specific melting temperature at 82.5°C for MAT1‑1 and at 84.0°C for MAT1-2, respectively.

# Supplementary Tables and Figure

## Supplementary Figures


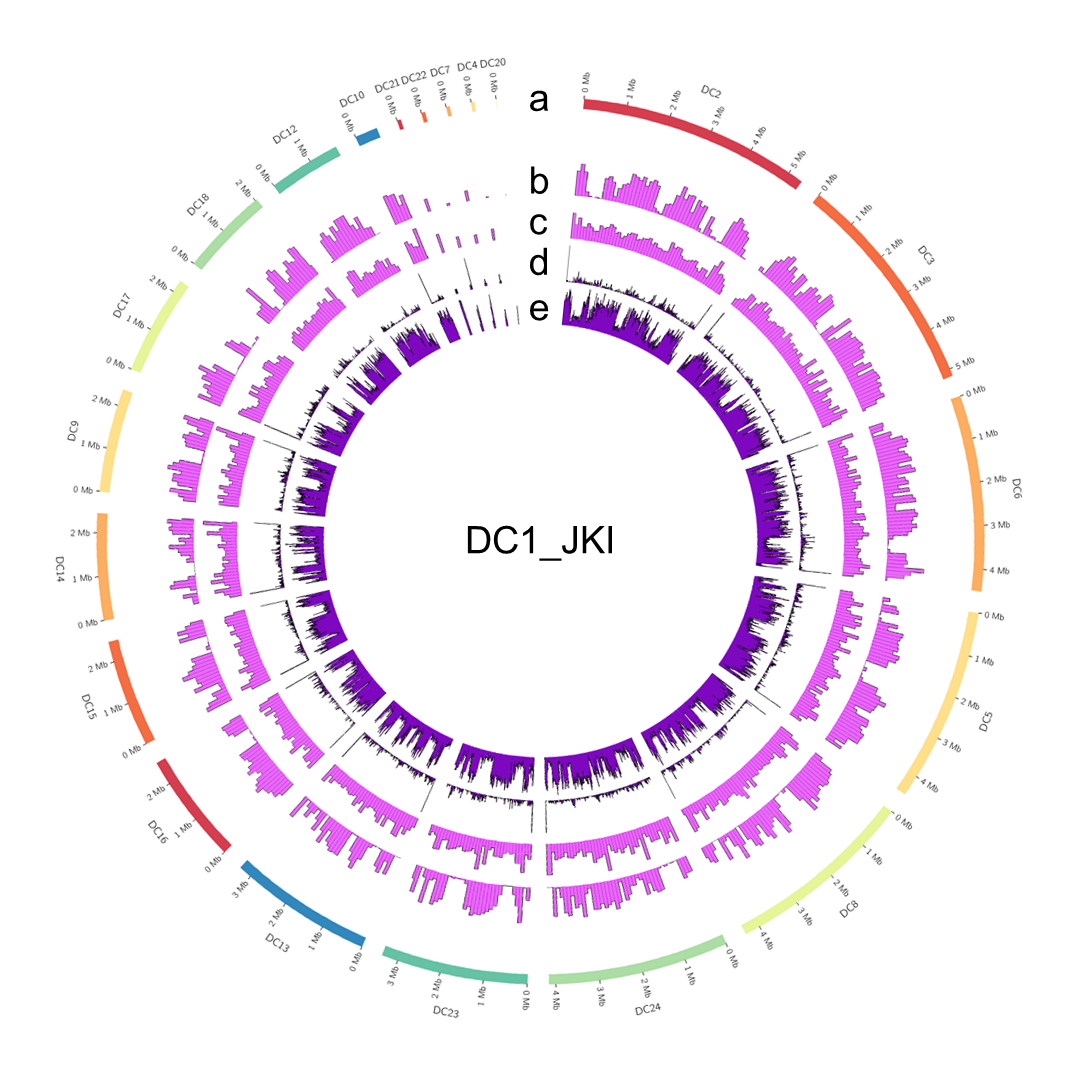
**Figure S1** The gapless t2t genome assembly of *D. coronariae* DC1_JKI. Circos plot of 21 pseudomolecules representing the chromosome constitution. DC19 represents the mitochondrial sequence of *D. coronariae.* (a) chromosome length (Mb); (b) gene density in blocks of 100k; (c) distribution of repetitive sequences in blocks of 100k; (d) telomere repeat distribution (TTAGGG, CCCTAA) in blocks of 10k indicating nine t2t, six partial t2t and six without telomere repeats assembled chromosomes at the chromosomal end; (e) GC content in blocks of 10k.


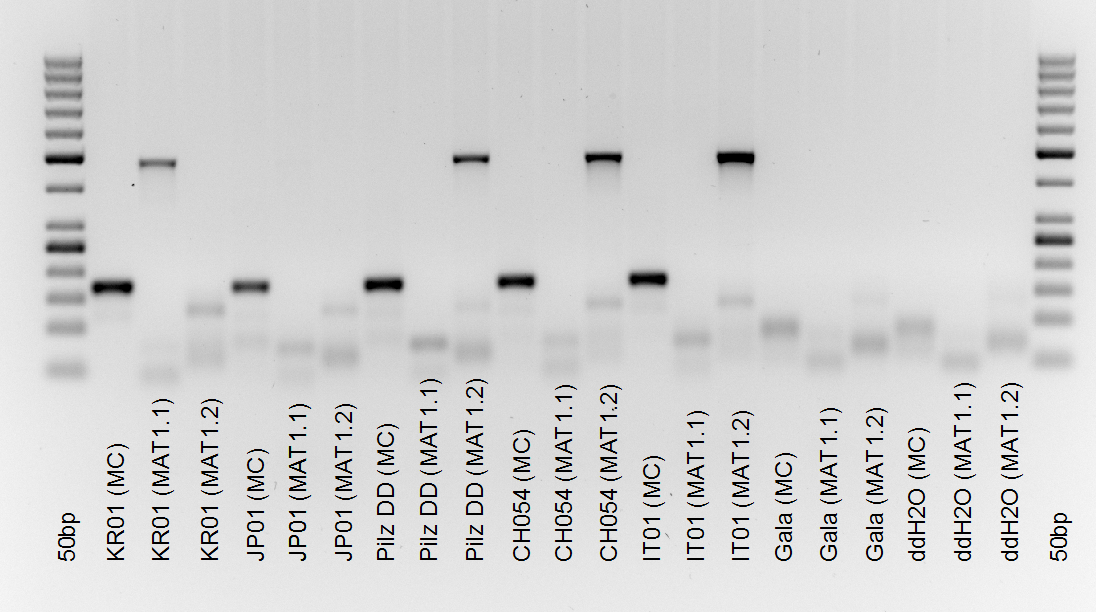


**Figure S2** Detection of mating 1.1 and mating type 1.2 of *Diplocarpon coronariae* in Asian and European isolates/leaf samples. KR01 – Korea; JP01 – Japan; Pilz DD – Germany; CH054 – Switzerland; Gala – symptom free apple leaf; ddH2O – DNA-free negative control; 50bp – size standard; MC – Primer for specific ITS region

## Supplementary Tables

**Table S1** Analyzed samples from this study.

| **Sample accession no.** | **Country** | **Region/Town** | **Year** | **Sample type** | **Cultivar** | **Multi-Locus Genotype** | **Reference** |
| --- | --- | --- | --- | --- | --- | --- | --- |
| NBRC 30405 | Japan | Aomori | 1977 | fungal isolate | *Malus pumila* var*. domestica* | MLG05 | Harada et al. (1974) |
| CH024 | Switzerland | Boll | 2016 | leaf sample | unknown | MLG38 | Oberhänsli et al. (2021) |
| DC_JKI1 | Germany | Dresden | 2016 | fungal isolate | unknown | unknown | this study |
| CH032 | Switzerland | Frick | 2016 | fungal isolate | unknown | MLG21 | Oberhänsli et al. (2021) |
| CH053 |  | Horgen | 2016 | leaf sample | unknown | MLG18 |  |
| CH052 |  |  | 2016 | leaf sample | unknown | MLG21 |  |
| CH054 |  |  | 2016 | leaf sample | unknown | MLG33 |  |
| CH001 |  | Morges | 2016 | leaf sample | unknown | MLG38 |  |
| CH002 |  |  | 2016 | leaf sample | unknown | MLG38 |  |
| CH033 |  | Seengen | 2016 | leaf sample | unknown | MLG31 |  |
| CH043 |  | Steinmaur | 2016 | leaf sample | unknown | MLG31 |  |
| DE67 | Germany | Bavendorf | 2017 | leaf sample | unknown | MLG11 |  |
| DE19 |  |  | 2017 | leaf sample | unknown | MLG12 |  |
| DE20 |  |  | 2017 | leaf sample | unknown | MLG12 |  |
| DE21 |  |  | 2017 | leaf sample | unknown | MLG12 |  |
| DE17 |  |  | 2017 | leaf sample | unknown | MLG17 |  |
| DE18 |  |  | 2017 | leaf sample | unknown | MLG17 |  |
| DE65 |  |  | 2017 | leaf sample | unknown | MLG20 |  |
| DE62 |  |  | 2017 | leaf sample | unknown | MLG23 |  |
| DE63 |  |  | 2017 | leaf sample | unknown | MLG23 |  |
| DE66 |  |  | 2017 | leaf sample | unknown | MLG33 |  |
| DE14 |  |  | 2017 | leaf sample | unknown | MLG36 |  |
| KR01 | Korea | Daegu | 2017 | leaf sample | unknown | MLG04 |  |
| CH025 | Switzerland | Frick | 2017 | leaf sample | unknown | MLG37 |  |
| IT01 | Italy | Gardasee | 2017 | leaf sample | unknown | MLG38 |  |
| IT02 | Italy | Gardasee | 2017 | leaf sample | unknown | MLG38 |  |
| CH018 | Switzerland | Hessigkofen | 2017 | leaf sample | unknown | MLG19 | Oberhänsli et al. (2021) |
| CH019 |  |  | 2017 | leaf sample | unknown | MLG19 |  |
| CH022 |  |  | 2017 | leaf sample | unknown | MLG21 |  |
| CH040 |  | Kerns | 2017 | fungal isolate | unknown | MLG38 |  |
| CH042 |  | Oberlunkhofen | 2017 | leaf sample | unknown | MLG34 |  |
| CH068 |  | Wädenswil | 2017 | fungal isolate | unknown | MLG34 |  |
| CH050 |  | Zürich | 2017 | leaf sample | unknown | MLG30 |  |
| KR06 | Korea | Uiseong County | 2017 | leaf sample | unknown | unknown | this study |
| KR07 |  |  | 2017 | leaf sample | unknown | unknown |  |
| KR08 |  |  | 2017 | leaf sample | unknown | unknown |  |
| KR09 |  |  | 2017 | leaf sample | unknown | unknown |  |
| KR10 |  |  | 2017 | leaf sample | unknown | unknown |  |
| KR11 |  |  | 2017 | leaf sample | unknown | unknown |  |
| KR28 |  |  | 2017 | leaf sample | unknown | unknown |  |
| KR29 |  |  | 2017 | leaf sample | unknown | unknown |  |
| KR30 |  |  | 2017 | leaf sample | unknown | unknown |  |
| KR44 |  | Sangju | 2017 | leaf sample | unknown | unknown |  |
| KR45 |  |  | 2017 | leaf sample | unknown | unknown |  |
| KR46 |  |  | 2017 | leaf sample | unknown | unknown |  |
| KR47 |  |  | 2017 | leaf sample | unknown | unknown |  |
| KR48 |  |  | 2017 | leaf sample | unknown | unknown |  |
| KR49 |  |  | 2017 | leaf sample | unknown | unknown |  |
| KR50 |  |  | 2017 | leaf sample | unknown | unknown |  |
| KR71 |  |  | 2017 | leaf sample | unknown | unknown |  |
| KR72 |  |  | 2017 | leaf sample | unknown | unknown |  |
| KR73 |  |  | 2017 | leaf sample | unknown | unknown |  |
| KR74 |  |  | 2017 | leaf sample | unknown | unknown |  |
| KR75 | Korea | Sangju | 2017 | leaf sample | unknown | unknown | this study |
| KR76 |  |  | 2017 | leaf sample | unknown | unknown |  |
| KR77 |  |  | 2017 | leaf sample | unknown | unknown |  |
| KR78 |  |  | 2017 | leaf sample | unknown | unknown |  |
| CAN1 | Canada | Compton, Québec | 2022 | leaf sample | Golden Russet | unknown |  |
| CAN2 |  |  | 2022 | leaf sample | Golden Russet | unknown |  |
| CAN3 |  |  | 2022 | leaf sample | Golden Russet | unknown |  |
| CAN4 |  |  | 2022 | leaf sample | Golden Russet | unknown |  |
| CAN5 |  |  | 2022 | leaf sample | Golden Russet | unknown |  |
| CAN6 |  |  | 2022 | leaf sample | Golden Russet | unknown |  |
| CAN7 |  |  | 2022 | leaf sample | Golden Russet | unknown |  |
| CAN8 |  |  | 2022 | leaf sample | Golden Russet | unknown |  |
| CAN9 |  |  | 2022 | leaf sample | Golden Russet | unknown |  |
| CAN10 |  |  | 2022 | leaf sample | Sunrise | unknown |  |
| CAN11 |  |  | 2022 | leaf sample | Sunrise | unknown |  |
| CAN12 |  |  | 2022 | leaf sample | Sunrise | unknown |  |
| CAN13 |  |  | 2022 | leaf sample | Sunrise | unknown |  |
| CAN14 |  |  | 2022 | leaf sample | Sunrise | unknown |  |
| CAN15 |  |  | 2022 | leaf sample | Sunrise | unknown |  |
| CAN16 |  |  | 2022 | leaf sample | Sunrise | unknown |  |
| CAN17 |  |  | 2022 | leaf sample | Sunrise | unknown |  |
| CAN18 |  |  | 2022 | leaf sample | Sunrise | unknown |  |
| CAN19 |  | Saint-Bruno, Québec | 2022 | leaf sample | Ginger Gold | unknown |  |
| CAN20 |  |  | 2022 | leaf sample | Ginger Gold | unknown |  |
| CAN21 |  |  | 2022 | leaf sample | Ginger Gold | unknown |  |
| CAN22 |  |  | 2022 | leaf sample | Ginger Gold | unknown |  |
| CAN23 |  |  | 2022 | leaf sample | Ginger Gold | unknown |  |
| CAN24 | Canada | Saint-Joseph-du-Lac, Québec | 2022 | leaf sample | Empire | unknown | this study |
| CAN25 |  |  | 2022 | leaf sample | Empire | unknown |  |
| CAN26 |  |  | 2022 | leaf sample | Empire | unknown |  |
| CAN27 |  |  | 2022 | leaf sample | Empire | unknown |  |
| CAN28 |  |  | 2022 | leaf sample | Empire | unknown |  |
| CAN29 |  |  | 2022 | leaf sample | Empire | unknown |  |
| CAN30 |  |  | 2022 | leaf sample | Empire | unknown |  |
| CH22-13 | France | Corcelles-Chamdor | 2022 | leaf sample | unknown | unknown |  |
| CH22-12 | Germany | Langenargen | 2022 | leaf sample | unknown | unknown |  |
| CH22-14 |  | Bavendorf | 2022 | leaf sample | unknown | unknown |  |
| RB-01 |  | Radebeul | 2022 | leaf sample | unknown | unknown |  |
| BY-01 |  | Kehlheim | 2022 | leaf sample | unknown | unknown |  |
| CH22-9 | Liechtenstein | Schaan | 2022 | leaf sample | unknown | unknown |  |
| CH22-6 | Switzerland | Einsiedeln | 2022 | leaf sample | unknown | unknown |  |
| CH22-2 |  | Flawil | 2022 | leaf sample | Golden Delicious | unknown |  |
| CH22-4 |  |  | 2022 | leaf sample | Topaz | unknown |  |
| CH22-8 |  | Gottshalden | 2022 | leaf sample | unknown | unknown |  |
| CH22-3 |  | Wädenswil | 2022 | leaf sample | Golden Delicious (Wa27) | unknown |  |
| CH22-10 |  |  | 2022 | leaf sample | unknown | unknown |  |
| CH22-11 |  |  | 2022 | leaf sample | unknown | unknown |  |
| CH22-1 |  | Winterthur | 2022 | leaf sample | Relinda (Wülo) | unknown |  |
| CH22-5 |  |  | 2022 | leaf sample | Topaz(Wülo) | unknown |  |
| CH22-7 |  |  | 2022 | leaf sample | Boskoop (Wü10) | unknown |  |
| JP22-1 | Japan | Shimokuriyagawa, Morioka city, Iwate prefecture | 2023 | leaf sample | unknown | unknown |  |
| JP22-2 | Japan | Shimokuriyagawa, Morioka city, Iwate prefecture | 2023 | leaf sample | unknown | unknown | this study |
| JP22-3 |  |  | 2023 | leaf sample | unknown | unknown |  |
| JP22-4 |  |  | 2023 | leaf sample | unknown | unknown |  |
| JP22-5 |  |  | 2023 | leaf sample | unknown | unknown |  |
| JP22-6 |  |  | 2023 | leaf sample | unknown | unknown |  |
| JP22-7 |  |  | 2023 | leaf sample | unknown | unknown |  |
| JP22-8 |  |  | 2023 | leaf sample | unknown | unknown |  |
| JP22-9 |  |  | 2023 | leaf sample | unknown | unknown |  |
| JP22-10 |  |  | 2023 | leaf sample | unknown | unknown |  |

**Table S2** RNA-Seq libraries of several species for spliced mapping

| *species* | *SRA number* |
| --- | --- |
| *Cadophora sp*. DSE1049 | SRR1587637 |
| *Drepanopeziza brunnea* | SRR6303469 |
|  | SRR6303465 |
|  | SRR6303466 |
|  | SRR6303468 |
|  | SRR6303470 |
|  | SRR6303467 |
| *Drepanopeziza brunnea f. sp. 'multigermtubi'* | SRR504340 |
| *Hymenoscyphus fraxineus* | ERR756144 |
|  | ERR756148 |
|  | ERR756147 |
|  | ERR756146 |
|  | ERR6323423 |
|  | ERR756145 |
| *Monilinia laxa* | SRR6312175 |
|  | SRR6312182 |
|  | SRR6312187 |
|  | SRR6312174 |
|  | SRR6312190 |
|  | SRR6312181 |
| *Rhynchosporium commune* | SRR17952721 |
|  | SRR17952720 |

**Table S3** Primer and their sequences used for standard PCR

| Region | Primer | Sequence 5´-3´ | Reference |
| --- | --- | --- | --- |
| ITS | Mc_Forward | GCC TAC CCT ACC TCT GTT GC | Oberhänsli *et al.* 2014 |
|  | Mc_Reverse | CAG AAC CAA GAG ATC CGT TGT TG | Oberhänsli *et* *al*. 2014 |
| Mating Locus | MAT1-1-specific_forward | ATTCGCAGTCTCATACGCTACAT | Cheng *et al*., 2021 |
|  | MAT1-1-specific_reverse | AGCCTTTGGAATCTTCTGCTGTA | Cheng *et al*., 2021 |
|  | MAT1-2-specific_forward | GAAGTTGAGTGGTCTATGAGCCA | Cheng *et al*., 2021 |
|  | MAT1-2-specific_reverse | CCCTACTTTACCCAGACTATGCC | Cheng *et al*., 2021 |

**Table S4** Number of syntenic blocks shared between contigs from *D. rosae* and *D. coronariae*

| *D. rosae* | *D. coronariae* | Syntenic blocks |
| --- | --- | --- |
| JAUBYV010000001.1 | tig00000003 | 3 |
| JAUBYV010000001.1 | tig00000005 | 5 |
| JAUBYV010000001.1 | tig00000006 | 3 |
| JAUBYV010000001.1 | tig00000009 | 2 |
| JAUBYV010000002.1 | tig00000006 | 1 |
| JAUBYV010000002.1 | tig00000007 | 1 |
| JAUBYV010000002.1 | tig00000012 | 1 |
| JAUBYV010000002.1 | tig00000018 | 2 |
| JAUBYV010000003.1 | tig00000002 | 5 |
| JAUBYV010000004.1 | tig00000013 | 3 |
| JAUBYV010000004.1 | tig00000024 | 3 |
| JAUBYV010000005.1 | tig00000003 | 3 |
| JAUBYV010000006.1 | tig00000012 | 2 |
| JAUBYV010000006.1 | tig00000014 | 2 |
| JAUBYV010000007.1 | tig00000010 | 1 |
| JAUBYV010000007.1 | tig00000023 | 4 |
| JAUBYV010000008.1 | tig00000008 | 3 |
| JAUBYV010000008.1 | tig00000017 | 2 |
| JAUBYV010000009.1 | tig00000016 | 4 |
| JAUBYV010000010.1 | tig00000008 | 3 |
| JAUBYV010000010.1 | tig00000017 | 3 |
| JAUBYV010000011.1 | tig00000024 | 2 |
| JAUBYV010000012.1 | tig00000015 | 3 |
| JAUBYV010000012.1 | tig00000018 | 1 |
| JAUBYV010000013.1 | tig00000015 | 1 |
| JAUBYV010000013.1 | tig00000017 | 1 |
| JAUBYV010000014.1 | tig00000023 | 1 |

Table S5 BLASTN results obtained after local alignment of MAT specific primers sequences from Cheng et al. (2021) for the assembly DC1_JKI. For each primer the parameters, hit sequence, E-value, score, HSP (high-scoring segment pair) start and end positions, HSP length, percentage identity, and percentage gap, are given and provide insights into the effectiveness and specificity of the primers.

| **Primer** | **Hit** | **E-value** | **Score** | **HSP start** | **HSP end** | **HSP length** | **%Identity** | **%Gaps** |
| --- | --- | --- | --- | --- | --- | --- | --- | --- |
| MAT1-1_forward | tig00000017 | 0.455649 | 31 | 177748 | 177765 | 18 | 94.44 | 0 |
|  | tig00000024 | 0.455649 | 30 | 258109 | 258123 | 15 | 100 | 0 |
|  | tig00000005 | 5.55094 | 27 | 3580844 | 3580859 | 16 | 93.75 | 0 |
|  | tig00000005 | 5.55094 | 26 | 1625771 | 1625783 | 13 | 100 | 0 |
|  | tig00000003 | 5.55094 | 26 | 1413185 | 1413197 | 13 | 100 | 0 |
| MAT1-1_reverse | tig00000018 | 1.59037 | 29 | 1735399 | 1735415 | 17 | 94.12 | 0 |
|  | tig00000013 | 1.59037 | 29 | 931604 | 931588 | 17 | 94.12 | 0 |
|  | tig00000008 | 1.59037 | 29 | 2019925 | 2019909 | 17 | 94.12 | 0 |
|  | tig00000008 | 5.55094 | 26 | 290632 | 290644 | 13 | 100 | 0 |
|  | tig00000008 | 5.55094 | 26 | 3016380 | 3016368 | 13 | 100 | 0 |
|  | tig00000006 | 1.59037 | 28 | 607536 | 607554 | 19 | 89.47 | 0 |
|  | tig00000006 | 5.55094 | 27 | 2648260 | 2648275 | 16 | 93.75 | 0 |
|  | tig00000003 | 1.59037 | 28 | 3498899 | 3498912 | 14 | 100 | 0 |
|  | tig00000003 | 5.55094 | 27 | 1638645 | 1638660 | 16 | 93.75 | 0 |
|  | tig00000003 | 5.55094 | 26 | 4297320 | 4297308 | 13 | 100 | 0 |
|  | tig00000005 | 5.55094 | 27 | 3433433 | 3433418 | 16 | 93.75 | 0 |
|  | tig00000005 | 5.55094 | 26 | 3811399 | 3811387 | 13 | 100 | 0 |
|  | tig00000017 | 5.55094 | 26 | 1831502 | 1831514 | 13 | 100 | 0 |
|  | tig00000015 | 5.55094 | 26 | 1248930 | 1248918 | 13 | 100 | 0 |
|  | tig00000009 | 5.55094 | 26 | 584190 | 584202 | 13 | 100 | 0 |
|  | tig00000009 | 5.55094 | 26 | 1066367 | 1066384 | 18 | 88.89 | 0 |
|  | tig00000002 | 5.55094 | 26 | 4613 | 4625 | 13 | 100 | 0 |
|  | tig00000002 | 5.55094 | 26 | 929012 | 929000 | 13 | 100 | 0 |
|  | tig00000002 | 5.55094 | 26 | 4685016 | 4684999 | 18 | 88.89 | 0 |
| MAT-1-2_forward | tig00000009 | 2.07E-05 | 46 | 941186 | 941208 | 23 | 100 | 0 |
| MAT-1-2_forward | tig00000024 | 5.55094 | 27 | 3097865 | 3097880 | 16 | 93.75 | 0 |
|  | tig00000016 | 5.55094 | 27 | 1549254 | 1549269 | 16 | 93.75 | 0 |
|  | tig00000013 | 5.55094 | 27 | 1101749 | 1101734 | 16 | 93.75 | 0 |
|  | tig00000013 | 5.55094 | 27 | 1101793 | 1101778 | 16 | 93.75 | 0 |
|  | tig00000003 | 5.55094 | 27 | 1634562 | 1634577 | 16 | 93.75 | 0 |
|  | tig00000006 | 5.55094 | 26 | 3707738 | 3707726 | 13 | 100 | 0 |
|  | tig00000002 | 5.55094 | 26 | 4593224 | 4593212 | 13 | 100 | 0 |
| MAT-1-2_reverse | tig00000009 | 2.07E-05 | 46 | 941661 | 941639 | 23 | 100 | 0 |
|  | tig00000018 | 0.455649 | 30 | 248666 | 248680 | 15 | 100 | 0 |
|  | tig00000017 | 5.550940 | 27 | 62998 | 62983 | 16 | 93.75 | 0 |
|  | tig00000003 | 5.550940 | 27 | 2028113 | 2028128 | 16 | 93.75 | 0 |
|  | tig00000003 | 5.550940 | 26 | 4820698 | 4820686 | 13 | 100 | 0 |
|  | tig00000006 | 5.550940 | 26 | 776072 | 776060 | 13 | 100 | 0 |
|  | tig00000005 | 5.550940 | 26 | 1445501 | 1445518 | 18 | 88.89 | 0 |

Table S6 BLASTN results obtained after local alignment of MAT specific primers sequences from Cheng et al. (2021) for the assembly NBRC_30405. For each primer the parameters, hit sequence, E-value, score, HSP (high-scoring segment pair) start and end positions, HSP length, percentage identity, and percentage gap, are given and provide insights into the effectiveness and specificity of the primers.

| Primer | Hit | E-value | Score | HSP start | HSP end | HSP length | %Identity | %Gaps |
| --- | --- | --- | --- | --- | --- | --- | --- | --- |
| MAT1-1_forward | tig00000469 | 2.04E-05 | 46 | 589665 | 589687 | 23 | 100 | 0 |
|  | tig00000313 | 0.448489 | 31 | 174465 | 174448 | 18 | 94 | 0 |
|  | tig00000281 | 0.448489 | 30 | 134869 | 134855 | 15 | 100 | 0 |
|  | tig00000798 | 5.463710 | 27 | 134202 | 134187 | 16 | 93.75 | 0 |
|  | tig00000496 | 5.463710 | 26 | 855684 | 855672 | 13 | 100 | 0 |
|  | tig00000365 | 5.463710 | 26 | 474453 | 474441 | 13 | 100 | 0 |
| MAT1-1_reverse | tig00000469 | 2.04E-05 | 46 | 590132 | 590110 | 23 | 100 | 0 |
|  | tig00000358 | 1.565380 | 29 | 302253 | 302237 | 17 | 94.12 | 0 |
|  | tig00000059 | 1.565380 | 29 | 178276 | 178260 | 17 | 94.12 | 0 |
|  | tig00000634 | 1.565380 | 28 | 167805 | 167823 | 19 | 89.47 | 0 |
|  | tig00000527 | 1.565380 | 28 | 1469141 | 1469128 | 14 | 100 | 0 |
|  | tig00000527 | 5.463710 | 26 | 798924 | 798936 | 13 | 100 | 0 |
|  | tig00000595 | 5.463710 | 27 | 248013 | 247998 | 16 | 93.75 | 0 |
|  | tig00000437 | 5.463710 | 27 | 152584 | 152569 | 16 | 93.75 | 0 |
|  | tig00000400 | 5.463710 | 27 | 149953 | 149968 | 16 | 93.75 | 0 |
|  | tig00000365 | 5.463710 | 27 | 248672 | 248657 | 16 | 93.75 | 0 |
|  | tig00000240 | 5.463710 | 27 | 13048 | 13063 | 16 | 93.75 | 0 |
|  | tig00000827 | 5.463710 | 26 | 136482 | 136470 | 13 | 100 | 0 |
|  | tig00000667 | 5.463710 | 26 | 59044 | 59027 | 18 | 88.89 | 0 |
|  | tig00000364 | 5.463710 | 26 | 230517 | 230529 | 13 | 100 | 0 |
|  | tig00000332 | 5.463710 | 26 | 309049 | 309037 | 13 | 100 | 0 |
|  | tig00000250 | 5.463710 | 26 | 220451 | 220434 | 18 | 88.89 | 0 |
|  | tig00000195 | 5.463710 | 26 | 512438 | 512426 | 13 | 100 | 0 |
| MAT-1-2_forward | tig00000606 | 5.463710 | 27 | 109015 | 109000 | 16 | 93.75 | 0 |
| MAT-1-2_reverse | tig00000770 | 0.448489 | 30 | 182968 | 182954 | 15 | 100 | 0 |
|  | tig00000512 | 5.463710 | 27 | 207589 | 207574 | 16 | 93.75 | 0 |
|  | tig00000313 | 5.463710 | 27 | 268606 | 268621 | 16 | 93.75 | 0 |
|  | tig00000634 | 5.463710 | 26 | 336394 | 336382 | 13 | 100 | 0 |
|  | tig00000613 | 5.463710 | 26 | 387999 | 388011 | 13 | 100 | 0 |
|  | tig00000496 | 5.463710 | 26 | 1035582 | 1035565 | 18 | 88.89 | 0 |

**Table S7** Detection of mating type 1-1 (Mat1-1) and mating type 1-2 (Mat1-2) in samples of *Diplocarpon coronariae*, as well as the Detection of the specific ITS region of the fungus (ITS)*.* The Multi-locus genotype according to Oberhänsli et al. 2021 is assigned to the relevant samples.

| Country | Multi-locus genotype | Sample | ITS | Mat 1-1 | Mat 1-2 |
| --- | --- | --- | --- | --- | --- |
| Switzerland | 38 | CH001 | + | - | (+) |
|  | 38 | CH002 | + | - | + |
|  | 19 | CH018 | + | - | + |
|  | 19 | CH019 | + | - | + |
|  | 21 | CH022 | + | - | + |
|  | 38 | CH024 | + | - | + |
|  | 38 | CH025 | + | - | + |
|  | 21 | CH032 | + | - | + |
|  | 31 | CH033 | + | - | + |
|  | 38 | CH040 | + | - | + |
|  | 34 | CH042 | + | - | + |
|  | 31 | CH043 | + | - | (+) |
|  | 30 | CH050 | + | - | + |
|  | 21 | CH052 | + | - | (+) |
|  | 18 | CH053 | + | - | (+) |
|  | 33 | CH054 | + | - | + |
|  | 34 | CH068 | + | - | (+) |
|  | n.a. | CH22-1 | + | - | + |
|  | n.a. | CH22-2 | + | - | (+) |
|  | n.a. | CH22-3 | + | - | + |
|  | n.a. | CH22-4 | + | - | + |
|  | n.a. | CH22-5 | + | - | + |
|  | n.a. | CH22-7 | + | - | (+) |
|  | n.a. | CH22-8 | + | - | + |
|  | n.a. | CH22-10 | + | - | + |
|  | n.a. | CH22-11 | + | - | + |
| Germany | 36 | DE14 | + | - | (+) |
|  | 17 | DE18 | + | - | (+) |
|  | 12 | DE20 | (+) | - | (+) |
|  | 12 | DE21 | + | - | + |
|  | 23 | DE62 | + | - | + |
|  | 33 | DE66 | + | - | (+) |
|  | n.a. | CH22-12 | + | - | + |
|  | n.a. | CH22-14 | + | - | + |
|  | n.a. | RB-01 | + | - | + |
|  | n.a. | DD | + | - | + |
|  | n.a. | BY | + | - | + |
|  | n.a. | DC1_JKI | + | - | + |
| Italy | 38 | IT01 | + | - | + |
|  | 38 | IT02 | + | - | + |
| Liechtenstein | n.a. | CH22-9 | + | - | (+) |
| France | n.a. | CH22-13 | + | - | + |

Continuation Table S7

| Country | Multi-locus genotype | Sample | ITS | Mat 1-1 | Mat 1-2 |
| --- | --- | --- | --- | --- | --- |
| Japan | 05 | JP01** | + | + | - |
|  | n.a. | JP22-1 | + | (+) | + |
|  | n.a. | JP22-2 | + | (+) | + |
|  | n.a. | JP22-3 | + | + | - |
|  | n.a. | JP22-4 | + | - | + |
|  | n.a. | JP22-5 | + | + | (+) |
|  | n.a. | JP22-6 | + | - | + |
|  | n.a. | JP22-7 | + | - | + |
|  | n.a. | JP22-8 | + | + | (+) |
|  | n.a. | JP22-9 | + | (+) | + |
|  | n.a. | JP22-10 | + | + | (+) |
| Korea | 04 | KR01 | + | + | - |
|  | n.a. | KR06 | + | (+) | - |
|  | n.a. | KR07 | + | (+) | - |
|  | n.a. | KR08 | + | (+) | - |
|  | n.a. | KR09 | + | (+) | (+) |
|  | n.a. | KR10 | + | + | - |
|  | n.a. | KR11 | + | (+) | (+) |
|  | n.a. | KR28 | + | (+) | - |
|  | n.a. | KR29 | + | + | - |
|  | n.a. | KR48 | + | - | + |
|  | n.a. | KR49 | + | (+) | - |
|  | n.a. | KR50 | + | + | (+) |
|  | n.a. | KR71 | + | - | (+) |
|  | n.a. | KR72 | + | (+) | (+) |
|  | n.a. | KR73 | + | (+) | (+) |
|  | n.a. | KR74 | + | (+) | - |
|  | n.a. | KR75 | + | (+) | - |
|  | n.a. | KR77 | + | (+) | - |
|  | n.a. | KR78 | + | (+) | - |
| Canada | n.a. | CAN01 | + | - | + |
|  | n.a. | CAN02 | + | - | + |
|  | n.a. | CAN03 | + | - | + |
|  | n.a. | CAN04 | + | - | + |
|  | n.a. | CAN05 | + | - | + |
|  | n.a. | CAN06 | + | - | + |
|  | n.a. | CAN07 | + | - | + |
|  | n.a. | CAN08 | + | - | + |
|  | n.a. | CAN09 | + | - | + |
|  | n.a. | CAN10 | + | - | + |

Continuation Table S7

| Country | Multi-locus genotype | Sample | ITS | Mat 1-1 | Mat 1-2 |
| --- | --- | --- | --- | --- | --- |
| Canada | - | CAN11 | + | - | + |
|  | - | CAN12 | + | - | + |
|  | - | CAN13 | + | - | + |
|  | - | CAN14 | + | - | + |
|  | - | CAN15 | + | - | + |
|  | - | CAN16 | + | - | + |
|  | - | CAN17 | + | - | + |
|  | - | CAN18 | + | - | + |
|  | - | CAN19 | + | - | + |
|  | - | CAN20 | + | - | + |
|  | - | CAN21 | + | - | + |
|  | - | CAN22 | + | - | + |
|  | - | CAN24 | + | - | + |
|  | - | CAN25 | + | - | + |
|  | - | CAN26 | (+) | - | (+) |
|  | - | CAN27 | + | - | + |
|  | - | CAN28 | + | - | + |
|  | - | CAN29 | + | - | + |
|  | - | CAN30 | + | - | + |
|  | 05 | Japan NBRC 30405** | + | + | - |
|  |  | Gala (apple leaf) | - | - | - |
|  |  | ddH2O | - | - | - |
|  |  |  |  |  |  |

+ presence of mating type

- absence of mating type

(+) weak banding and presence of mating type

** identical with Jp01 (MLG 05) and NBRC 30405
